# Engineered smart materials for RNA based molecular therapy to treat Glioblastoma

**DOI:** 10.1016/j.bioactmat.2023.11.007

**Published:** 2023-11-27

**Authors:** Ravi Raj Singh, Indranil Mondal, Taskeen Janjua, Amirali Popat, Ritu Kulshreshtha

**Affiliations:** a Department of Biochemical Engineering and Biotechnology, Indian Institute of Technology Delhi, New Delhi, India; b School of Pharmacy, The University of Queensland, Brisbane, QLD, 4072, Australia; c Department of Functional Materials and Catalysis, Faculty of Chemistry, University of Vienna, Währinger Straße 42, 1090 Vienna, Austria; d University of Queensland –IIT Delhi Academy of Research (UQIDAR)

**Keywords:** Glioblastoma (GBM), Non-coding RNAs (ncRNA), Nucleic acid therapy, Oligonucleotide therapy, Nanocarriers, Nanoparticles, RNA delivery system, Gene therapy

## Abstract

Glioblastoma (GBM) is an aggressive malignancy of the central nervous system (CNS) that remains incurable despite the multitude of improvements in cancer therapeutics. The conventional chemo and radiotherapy post-surgery have only been able to improve the prognosis slightly; however, the development of resistance and/or tumor recurrence is almost inevitable. There is a pressing need for adjuvant molecular therapies that can successfully and efficiently block tumor progression. During the last few decades, non-coding RNAs (ncRNAs) have emerged as key players in regulating various hallmarks of cancer including that of GBM. The levels of many ncRNAs are dysregulated in cancer, and ectopic modulation of their levels by delivering antagonists or overexpression constructs could serve as an attractive option for cancer therapy. The therapeutic potential of several types of ncRNAs, including miRNAs, lncRNAs, and circRNAs, has been validated in both *in vitro* and *in vivo* models of GBM. However, the delivery of these RNA-based therapeutics is highly challenging, especially to the tumors of the brain as the blood-brain barrier (BBB) poses as a major obstacle, among others. Also, since RNA is extremely fragile in nature, careful considerations must be met while designing a delivery agent. In this review we have shed light on how ncRNA therapy can overcome the limitations of its predecessor conventional therapy with an emphasis on smart nanomaterials that can aide in the safe and targeted delivery of nucleic acids to treat GBM. Additionally, critical gaps that currently exist for successful transition from viral to non-viral vector delivery systems have been identified. Finally, we have provided a perspective on the future directions, potential pathways, and target areas for achieving rapid clinical translation of, RNA-based macromolecular therapy to advance the effective treatment of GBM and other related diseases.

## Highlights

• Challenges faced by conventional GBM therapy with poor survival.
• Promises of RNA-based therapeutics, targeting critical genes and ncRNAs in GBM.
• Engineered nanomaterials aid in targeted delivery of therapeutic RNA to GBM tumor.
• Insights on the choice of suitable nanomaterial and therapeutic RNA for GBM therapy.
• Obstacles to translate RNA-based GBM therapy from bench to the bedside.

## Graphical abstract

**Figure:**
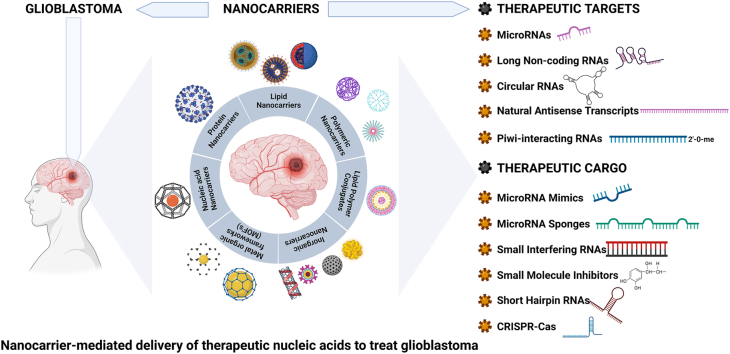

## 1 Introduction

Glioblastoma (GBM) is the most common and lethal primary malignancy of the central nervous system (CNS) having a dismal prognosis. The 2021 World Health Organization (WHO) classification of CNS tumors categorizes GBM as a grade IV diffuse glioma having the wild type allele of the isocitrate dehydrogenase (IDH) gene, while the IDH-mutant variant has been termed as ‘Astrocytoma, IDH-mutant’ [1]. Each year, on an average, about 3–4 GBM cases are reported per 100,000 persons in the developed countries. Although GBM has a low incidence rate, patients who are diagnosed with this disease usually have a median overall survival of 14–15 months [2]. The conventional method of treatment is maximal surgical resection, followed by concomitant chemo and radiotherapy. This therapeutic modality has prevailed for the last decade, and no significant breakthrough has occurred in GBM treatment. Patients usually develop resistance to temozolomide (TMZ), the most common chemotherapeutic drug, and the chances of tumor recurrence are also high. Recently, cancer immunotherapy has shown promise in the treatment of GBM. The anti-VEGF monoclonal antibody bevacizumab has been approved by the US-FDA for treatment of GBM [3]. Also, chimeric antigen receptor (CAR)-T cell-based immunotherapy has shown promises [[4], [5], [6]]. However, neither of them has significantly improved the mortality of GBM patients. These challenges warrant for a better, multimodal therapeutic approach that can target GBM progression at the molecular level and stop this insidious disease.

During the last couple of decades, non-coding RNAs (ncRNAs) that are not involved in coding for proteins have been established as crucial regulators of cancer onset and progression [[7], [8], [9]]. Among the various ncRNAs playing regulatory roles in the human genome, microRNAs (miRNAs), and long non-coding RNAs (lncRNAs) have been studied the most. The physiological levels of the above ncRNAs have been found to be dysregulated in various cancers as compared to respective healthy tissues. miRNAs regulate gene expression by binding to the mRNAs of target genes through complementary (imperfect) base pairing, followed by transcript degradation or inhibition of translation [10]. While, lncRNAs have a wider targetome; i.e. they may bind to miRNAs, mRNAs, DNA, and even proteins, thereby regulating gene expression [11]. Among the other regulatory ncRNAs, circular RNAs (circRNAs), piwi-interacting RNAs (piRNAs), natural antisense transcripts (NATs) are notable as they are deregulated in GBM and play either oncogenic or tumor suppressive roles [[12], [13], [14]]. Overall, these ncRNAs create a complex network interacting with each other and regulate GBM progression at the transcriptional, post-transcriptional, and epigenetic levels. Their altered expression in GBM and association with patient prognosis also highlight potential roles as diagnostic, and prognostic biomarkers. Overall, this class of RNAs are interesting therapeutic targets for GBM treatment, and successfully delivering their antagonists or agonists to tumor site may prove to be a powerful adjunct tool along with chemo/radiation therapy.

Despite the innumerable promises that ncRNA therapy for GBM might hold, the major bottleneck that prevents it from being translated to the bedside is the lack of efficient delivery of these RNA-based therapeutics to the tumor site. The harsh conditions offered by the delivery routes and tumor microenvironment make the RNA-based therapeutics susceptible to degradation by serum RNases, and rapid clearance by the reticuloendothelial system [15,16]. In addition, the blood-brain barrier (BBB) poses as a major obstacle for the delivery of >98 % potential therapeutics to the brain [17]. Given these therapeutic obstacles, multiple research groups have developed nanomaterials that can deliver these therapeutics to the tumor site while protecting them from nucleases, polyanionic moieties, etc. These nanocarriers also offer unique advantages such as low cytotoxicity, ability to cross the BBB, stealth to bypass immune detection, prolonged release of the cargo, improved circulatory time, and also targeted therapy. However, each of these smart RNA-nanocarrier has its own limitations.

In this review, we have attempted to offer a comprehensive overview of ncRNA-based therapeutics for GBM, discussed the potentials and limitations of various engineered nanomaterials for their delivery, and also proposed possible solutions to the therapeutic challenges.

## 2 Conventional GBM therapy regimen and their limitations

GBM is one of the most common, aggressive, lethal, and malignant forms of CNS tumor with high mortality, poor prognosis, and complex heterogeneity [1,2,18]. It is classified based on the IDH-wildtype gene, which is also used as a lineage marker for gliomagenesis [1,19,20]. Clinically, GBM is primarily located in the cerebral hemisphere and is rarely found in the spinal cord and cerebellum [21]. The annual incidence of GBM globally is between 0.59 and 5 cases per 100,000 persons and 3–4 cases per 100,000 persons in developed nations [2,22]. Age and sex are two key factors in GBM pathogenesis. In adults, GBM is diagnosed at a median age of 64, with the highest incident rates at 75–84 years, whereas pediatric GBM accounts for only 3–5% of all brain tumors in children [18,23]. Males are predominantly affected by GBM compared to women, with an M/F ratio ranging from 1.33 to 1.5 in patients diagnosed with primary GBM and a range of 0.65–2.3 in patients diagnosed with secondary GBM [24].

**Fig. 1):**
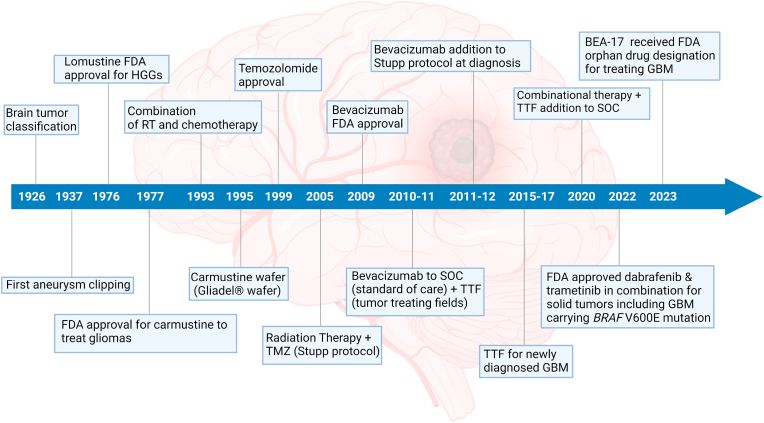
GBM therapy regimen evolution timeline. The field evolved with the very first classification of brain tumors by Bailey and Cushing followed by aneurysm clipping performed in 1937 by Walter E. Dandy on a patient diagnosed with Glioblastoma Multiforme. Later on, several developments came to treat GBM such as chemotherapy, radiotherapy, combination of chemo & radiotherapy. The first FDA approval for treating high-grade gliomas (HGGs) was granted to chemotherapeutic drugs lomustine and carmustine in the year 1976 and 1977 [71]. Then came the oral drug TMZ which got its FDA approval in the year 1999 for the treatment of anaplastic astrocytoma followed by the Stupp protocol in 2005 [25,37]. Roger Stupp in 2005 did a successful phase 3 clinical trial study where he and his team included radiotherapy alone and radiotherapy given concomitantly with adjuvant TMZ as standard of care for newly diagnosed GBM [25]. TMZ become the first line of chemotherapeutic drug to treat GBM. Treatment strategy was further advanced with the addition of bevacizumab to STUPP protocol for the treatment of GBM at the time of diagnosis in 2011–12 [72]. GBM therapy regimen was further improvised with the invention of tumor treating field and its addition to standard of care for GBM patients in 2015–2020 [67,69,70]. On June 2022, FDA approved dabrafenib and trametinib to be used in combination to treat solid tumors, including GBM, in patients with *BRAF* V600E mutation [73]. The drugs work in combination by inhibiting MAPK pathway enzymes such as MEK1 & MEK2 by trametinib and inhibition of B-Raf enzyme by dabrafenib [74]. Recently in February 2023, Beactica Therapeutics AB, a Sweden-based precision oncology company, received orphan drug designation for BEA-17 from the FDA to treat GBM [75]. BEA-17 is a small molecule that acts as a scaffold inhibitor by not only targeting epigenetic enzyme lysine-specific demethylase 1 (LSD1) but also simultaneously blocking the activity of its co-factor CoREST complex [76]. Figure created with BioRender.com

The mainstream therapy regimen for GBM is surgery and radiation followed by chemotherapy (Fig. 1) [[25], [26], [27]]. Primarily during surgery, the surgeon removes as much tumor as possible restricting damage to nearby tissues of the brain responsible for different neurological functions affecting motor or cognitive responses. Gliomas are generally surrounded by a class of migrating and infiltrating tumor cells, making it impossible to remove the tumor entirely [[28], [29], [30]]. Therefore, surgery only provides the option to reduce the amount of solid tumor in the brain, thereby prolonging patient survival by reducing intracranial pressure [31,32]. Another treatment regimen to treat GBM consists of radiation therapy performed after surgery when the wound is healed with a major goal to selectively kill tumor cells that have infiltrated to surrounding normal brain tissue. In order to treat infiltrating tumor cells, present in and around the zone, multiple fractions of radiation doses are given in a standard external beam therapy regimen [33,34]. The disadvantage of this is damage to the surrounding healthy microenvironment during treatment [35]. Depending upon the type of tumor, the treatment criteria of radiation are 10–30 cycles administered once a day or five times a week [33]. Radiotherapy is not often used as a primary treatment strategy in the case of GBM and is preferred for tumor showing recurrence [36].

Chemotherapy is also considered another treatment option for GBM combining surgery and radiotherapy. Currently, there are only three FDA approved therapies consisting use of TMZ, carmustine wafer (Gliadel® wafer) and bevacizumab; out of these, two are chemotherapeutic agents whereas bevacizumab is anti-VEGF therapy (targeted therapy) (Fig. 1) [3,25,37,38]. TMZ, which is currently used to treat GBM, is the first-line FDA approved chemotherapeutic drug [25,37,39]. TMZ acts as a monofunctional alkylating agent, methylating single strands of DNA at specific location mainly N^7^ and O^6^ region of guanine and N^3^ position of adenine [[39], [40], [41], [42]]. But due to the heterogeneous cell population as well as mutagenic nature of GBM along with extreme exposure to TMZ results in TMZ-resistance GBM which is a growing concern. Carmustine wafer, another FDA-approved therapy for GBM, belongs to nitrosourea group of alkylating agents which acts by forming O^6^ chloroethylguanine adducts [43]. It has been shown to only partially increase the overall survival (OS) with reduced mortality of only ∼30 % due to adverse events post-treatment [44]. Similary, adverse events post bevacizumab monotherapy or concomitant with other therapy regimen has also led to treatment discontinuation [45,46].

Apart from these, there are several other challenges to the conventional GBM therapy, e.g., peritumoral edema, intracranial hypertension, induction of tumor responsive seizures, neuronal toxicity, and tumor cells metastasizing locally throughout the CNS [[47], [48], [49]]. Therefore, there is an urgent need to focus on novel therapeutic strategies to target GBM.

## 3 Advanced therapeutic strategies to treat GBM

Several novel treatments are emerging for GBM therapy. One promising area of focus is RNA therapeutics, although currently there are relatively few of these therapies in clinical trials. Nevertheless, RNA therapeutics have the potential to be a game-changer in the future of targeted therapy for GBM. In this section, the promising therapies are evaluated with special focus on RNA-based therapies.

### 3.1 Immunotherapy

Numerous strategies have been developed using immune response to selectively target and destroy tumor cells. The 4 major immunotherapy-based treatment strategies that are currently in clinical trials to treat GBM include; 1) immune checkpoint inhibitors (CPI's); 2) modified CAR-T therapy; 3) oncolytic virus therapy and; 4) vaccine therapy [50]. Treatment strategies fail due to the inability of chemotherapeutic drugs to cross BBB, but reports suggest that CPI's such as nivolumab, durvalumab, pembrolizumab, pidilizumab and atezolizumab has shown promising results in treating other cancers with enhanced permeation ability towards BBB [[50], [51], [52]]. Similarly, in modified or engineered CAR-T immunotherapy, the T-cells are modified to express chimeric antigen receptors (CARs), which is an emerging therapeutic strategy against GBM [50]. Since, there is no FDA-approved T-cell therapy against GBM due to its extensive tumor heterogeneity; therefore, it would be significant to use it as a combination therapy rather than as a single therapy to further escalate its clinical translation. Recently, CAR T-cells are being employed for targeting receptors such as EGFR, CD70, EphA2, HER2 and interleukin-12 receptor-α [50,53].

In oncolytic virus therapy, the oncolytic viruses are employed to target tumor cells by mechanisms that consist of either direct oncolysis of tumor cells or by virus-induced anti-tumor immunity [50,54]. Oncolytic viruses have been proven to be advantageous by exerting an immunostimulatory effect [55]. But, the highly immunosuppressive GBM microenvironment is a major hurdle for designing novel oncolytic viral therapy regimens for GBM [56]. The results obtained in patients are far better by multimodal therapy approach combining immunotherapy with radiotherapy and chemotherapy rather than monotherapy alone. To date, there is no FDA-approved treatment for GBM using oncolytic viruses (viral construct).

Another form of active immunotherapy is vaccine therapy which helps in stimulating an immune response against various cancer-related antigens [57]. They are further categorized as cell-based and non-cell-based vaccines. The former is also referred to as patient-derived dendritic cell vaccine or autologous tumor cell vaccines, whereas; the latter is named peptide and heat shock protein vaccines. The peptide derived vaccines consist of sequences specifically engineered to induce targeted immunity against major histocompatibility complex (MHC) associated tumor antigens. This complex of MHC-associated tumor antigen is co-delivered with an immunostimulant adjuvant for cross-expression of antigen [[56], [57], [58]]. Other tumor vaccines include heat shock proteins that generate a highly responsive anti-tumor inflammatory response [57].

Similarly, autologous tumor cell vaccines are developed where patient-derived tumor cells are induced with cytotoxic T (Tc) Lymphocytes that are later re-administered in patients for generating anti-tumor immune response [57]. Cell-based vaccines such as dendritic cell vaccines targets glioma cells by activating CD8^+^ and CD4^+^ T cells for inducing tumor cell death. In this vaccine type, dendritic cells are obtained from the patients, loaded with glioma cell antigens, and then they are re-introduced inside the patient [57,58]. This immunotherapy approach provides the advantage of systemic specificity and negligible toxicity.

### 3.2 Laser interstitial thermal therapy (LITT)

Also referred to as laser-induced thermotherapy, this therapeutic modality offers GBM patients with the option to destroy tumor cells with increasing temperature. LITT is a minimally invasive, stereotactically guided neurosurgery technique. It is performed by implanting a fiberoptic laser catheter onto the tumor using a computer-guided navigation system followed by the selective ablation of target tumor tissue using heat energy generated from the laser [59]. It is usually opted when surgery is not possible due to the risk of cognitive impairment [60]. As far as thermal therapy is concerned, numerous treatment options such as ultrasound, radiofrequency, microwave or magnetic nanoparticle targeted delivery can be employed giving an advantage of minimal invasiveness [61]. Reports suggest MRI-guided LITT can disrupt peritumoral BBB enhancing therapeutic permeability [[62], [63], [64]]. It is also considered an alternative treatment strategy to surgery in recurrent GBM [65]. The patient can be discharged within 24-h post-treatment [66].

### 3.3 Tumor treating fields (TTF)

In this technology, low electric fields of intensity (1–3 V/cm) with a frequency of (100–300 kHz) are generated by placing non-invasive electrode arrays patched on the patient's head. Thus, applying a biophysical force by alternating electric fields affects different biological processes in cancer cells, including but not limited to DNA repair, anti-mitotic effects, autophagy, anti-migratory potential, antitumor immunity and changes in cell membrane permeability [67,68]. Novocure designed the FDA approved device Optune® in 2011 and its second generation in 2015, which is a functional example of TTF. For newly diagnosed GBM, it is used along with chemotherapy, whereas, in recurrent GBM, its use is intended as monotherapy (Fig. 1) [67,69]. Clinical trials showed the use of TTF combined with TMZ chemotherapy induced increase in survival rate from 16 months to 20.9 months without imposing any adverse effect on patient health [67,70].

## 4 RNA therapeutics to target GBM

The whole human genome sequencing revealed that only less than 3 % of the complete human genome codes for mRNA, which is then translated to proteins, while more than 90 % of the transcribed RNA encodes for ncRNAs [[77], [78], [79], [80]]. ncRNAs can either directly or indirectly control different cellular processes. They are not junk RNAs as previously thought; instead, the latest evidence suggests that they play a vital role in regulating different physiological, biological, and pathological processes [81].

**Fig. 2:**
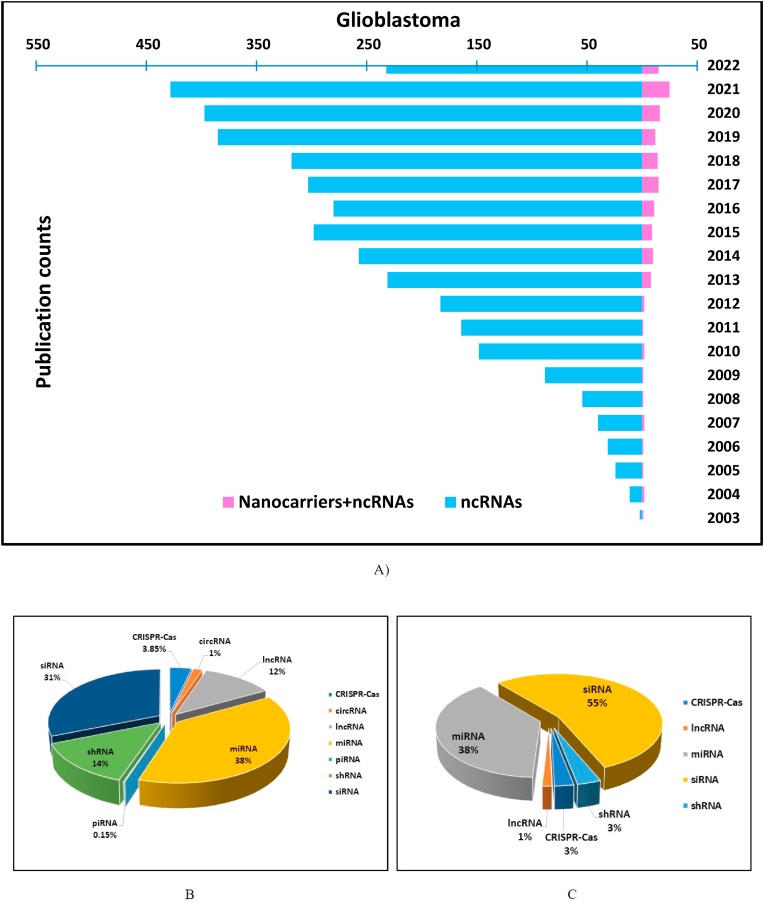
Comparative data from PubMed search of total number of publications in GBM involving ncRNAs & nanocarrier mediated delivery of therapeutics nucleic acids; A) Year wise number of publications in GBM related to ncRNAs and nanocarrier mediated delivery of nuclei acids, B) Percentage segregation of number of publications of different types of ncRNAs in GBM, C) Percentage segregation of number of studies involving nanocarrier mediated delivery & targeting ncRNAs. PubMed search keywords: ((Nanotechnology OR nanocarriers OR nanoparticles OR nanotherapeutics) AND (Glioblastoma OR GBM OR glioblastoma multiforme) AND (piRNAs OR Piwi interacting RNA OR siRNA OR small interfering RNA OR short interfering RNA OR interfering RNA OR shRNA OR small hairpin RNA OR short hairpin RNA OR MicroRNA OR miRNA OR miRs OR lncRNA OR long non-coding RNA OR long ncRNA OR CircularRNA OR circRNA OR CRISPR-CAS OR CRISPR-CAS9 OR CRISPR-CAS13 OR sgRNA OR non-coding RNAs OR ncRNAs)). Search criteria: Title/Abstract with manually entering the keywords followed by OR-AND format. The search was done in December 2022.

In cancers, such as GBM, depending on the role the ncRNAs play in tumorigenesis, they are classified into two main categories: oncogenic ncRNAs and tumor suppressor ncRNAs [82]. However, depending on the tumor and tissue type, they can act as both oncogenic and tumor suppressors [83]. In general, ncRNAs are classified as either long non-coding RNAs (lncRNAs), Circular RNAs (circRNAs) and small ncRNAs such as transfer RNAs (tRNAs) and ribosomal RNAs (rRNAs) whose biological functions are very well known. Whereas, there are other small ncRNAs such as small interfering RNAs (siRNAs), microRNAs (miRNAs), PIWI-interacting RNAs (piRNAs), small nuclear RNAs (snRNAs), small enhancer RNAs (seRNAs), small nucleolar RNAs (snoRNAs), small cajal body-specific RNAs (scaRNAs), telomerase RNA components (TERC) and Y RNAs, which are still under investigation to establish their clinical significance in tumor and normal cellular processes [77,[84], [85], [86], [87], [88], [89], [90], [91]]. The yearly publication data from PubMed search revealed that there had been approximately 4000 publications related to the study of ncRNAs in GBM, which is even less than 10 % of the total publications related to GBM since 1921. It is also surprising to note that even in studies pertaining to ncRNAs in GBM, studies involving the nanocarrier-mediated delivery of therapeutic nucleic acid to treat GBM is under 200, which is less than 5 % when compared to studies related to ncRNAs in GBM (Fig. 2). It was only after the advancement in nanotechnology since 2000 that researchers started to focus on using non-viral vectors to deliver therapeutics nucleic acids to treat GBM. This list of ncRNAs continues to grow with more and more advancements in genome sequencing. Out of this, small ncRNAs such as siRNA and miRNAs are already in clinical stages to treat GBM, but none is approved by the FDA or the European medicine agency. We have mainly focused on miRNA, lncRNA, circRNA, piRNAs and NATs in the current review.

### 4.1 microRNAs (miRNAs)

miRNAs are short, endogenously transcribed ncRNAs with a mature sequence length of ∼18–22 nucleotides, that inhibit gene expression by binding to messenger RNA, leading to transcript degradation, or inhibition of translation [92,93]. It is interesting to note that multiple miRNAs can regulate a single gene, and a single miRNA can regulate the expression of multiple genes [94,95]. While miRNAs have been generally shown to repress or downregulate the function of their target mRNA but there are reports where miRNAs have also been shown to promote or upregulate the expression of their target genes [96]. Expression profiles of miRNAs are known to be altered in several cancers. The aberrations/alterations in miRNAs expression can result from numerous mechanisms, including defects in miRNA localization, abnormalities during pri/pre miRNA processing, alterations in transcriptional regulation, mutations in miRNA encoding genes, and epigenetic alterations as CpG island methylation [97,98]. Specific miRNAs have been shown to play oncogenic/tumor suppressive roles by targeting various genes associated with cancer initiation/progression and thus regulate different hallmarks of cancers, such as enhancing cell proliferation, resisting cell death, autophagy, enabling replicative immortality, activating invasion and metastasis, and inducing angiogenesis [[99], [100], [101], [102], [103], [104], [105], [106], [107]]. Some of the prominent miRNAs associated with GBM pathogenesis have been categorized into tumor suppressors or oncogenic miRNAs and discussed below.

#### 4.1.1 Role of miRNAs in GBM

Out of the pool of deregulated tumor suppressor miRNAs, specific tumor suppressor miRNAs such as miR-7, miR-34a and miR128 are studied in detail and reported in different studies in context to GBM. miR-7, one of the most prominent tumor suppressor miRNAs in GBM, is known to be downregulated in GBM patients and has been shown to inhibit cellular proliferation, invasion, and metastasis [[108], [109], [110]]. miR-7 was shown to target multiple oncogenes and thus interfering the PI3K/Akt and EGFR pathways [110]. Similarly, miR-34a and miR-128 are other tumor suppressor miRNAs known to be downregulated and highly studied in context to GBM. The major targets of miR-34a and miR-128 in context to GBM as reported in numerous studies include SIRT1, c-Met, NOTCH1 and NOTCH2, PDGFRα, *MSI1*, Akt and Wnt for miR-34a; and P70S6K1, *SUZ12*, *Bmi-1-*, PDGFRα, EGFR, E2F-3a, *WEE1* and *MSI1* for miR-128 [[111], [112], [113], [114], [115], [116], [117], [118], [119], [120], [121], [122], [123], [124], [125], [126]].

The oncogenic miRNAs, also referred to as oncomiRs are highly overexpressed in GBM and in general target the tumor suppressor genes. Some of the most studied oncomiRs in GBM are miR-10b, miR-21 and miR-93. It was found that in majority of the high-grade gliomas, miR-10b expression was higher as compared to normal tissues [127,128]. The different targets of miR-10b reported in several studies cover HOXD10, uPAR, RhoC, CDKN1A, CDKN2A, PTEN, TFAP2C and BCL2L11 [[129], [130], [131], [132]]. The study conducted by Guessous and colleagues showed that inhibition of miR-10b using antisense approach significantly reduced the proliferation, invasion, and migration of GBM cells. *In-vivo* studies in orthotopic GBM xenografts also confirmed the same [131].

Similarly, ample evidence suggests that miR-21 and miR-93 also play a role in chemoresistance other than regulating proliferation, invasion, migration, and metastasis [100,107,[133], [134], [135]]. Of these two, miR-21 is known to be a major player in drug resistance. Shi et al., in their study, concluded that overexpression of miR-21 significantly reduced the efficacy of TMZ in U87-MG cells by downregulating the expression of Caspase-3, Bax and Bcl-2 [136]. Similarly, in other studies, downregulating the expression of miR-21 in GBM cells made them more sensitive to different chemotherapeutic drugs such as TMZ, paclitaxel, sunitinib and doxorubicin [[137], [138], [139], [140]]. The major targets of miR-21 that regulate different hallmarks of GBM include HNRNPK, PDCD4, p53, TAp63, TGF-β, Ras/Raf, ERKs, MMPs, PTEN, Spry2, SMARCA4, ANP32A and LRRFIP1 [103,105,106,137,[141], [142], [143], [144], [145]].

#### 4.1.2 miRNA-based target therapies to treat GBM

As discussed above, miRNAs play a dual role as either oncomiRs or tumor suppressor miRNAs in cancer progression. Depending on the role that they play in tumorigenesis, two different approaches can be devised to target the aberrantly expressed miRNAs in tumors: 1) If it is a tumor suppressor miRNA that is downregulated, then we can design miRNA mimics (synthetic miRNA analogs) to restore its function or 2) we can design miRNA inhibitors to inhibit the expression of oncomiRs that are upregulated.

##### 4.1.2.1 miRNA mimics

miRNA mimics therapy in GBM is aimed to target the downregulated tumor suppressor miRNAs to either restore the loss of function of a particular miRNA or to increase their tumor suppressor activity. The overexpression of tumor suppressor miRNAs can lead to downregulation of their target oncogenes which can inhibit tumor progression. This has been proven in several studies in GBM. In their research with U251 human GBM cells, Chen and colleagues showed that transfection of these cells with miR-203 synthetic analogs downregulated the expression of phospholipase D2, a known target of miR-203 [146]. Apart from miR-203, the levels of several other tumor suppressor miRNAs have been restored using miRNA mimics [15,118,147,148]. However, some groups have suggested that transient transfection using miRNA mimics may lead to the accumulation of high molecular weight RNA species in the cells, and hence, should be used with caution [149]. In such scenarios, transfection with recombinant plasmids or lentiviral constructs are preferred.

##### 4.1.2.2 AntagomiRs/anti-miRs

Antisense oligonucleotide therapeutics, also called anti-miRs or antagomiRs, are designed to target upregulated intracellular oncomiRs. These are synthetic oligonucleotides that bind to endogenous, mature miRNAs to repress their function. Usually, they are designed to be perfectly complementary to the seed regions of the target miRNAs and are conferred with chemical modifications to improve their efficacy and stability. Mostly, two types of modifications are introduced into the anti-miRs: sugar base modifications, and backbone modifications that protect them from degradation by nucleases. Among the important sugar modifications, 2′-O-methyl (2′-OMe), 2′-O-methoxyethyl (2′-MOE), 2′-Fluoro (2′-F), locked nucleic acid (LNA), and unlocked nucleic acid (UNA) are notable. While among the backbone modifications, peptide nucleic acid (PNA), phosphorothioate (PS), phosphonodiamidite morpholino oligomers (PMO), and phosphonoacetate (PACE) are noteworthy [150]. Song and colleagues designed antisense oligonucleotides to target miR-21, one of the most widely investigated oncomiRs in GBM. They coupled their antimiR with the R3V6 peptide, which protected this antimiR against cleavage by nucleases and enhanced cellular uptake. The R3V6 coupled antimiR was able to downregulate the expression of miR-21 and inhibit oncogenesis in GBM cells by inducing apoptosis [151]. This study was also supported by Oh and his group, where they delivered the antimiR against miR-21 using the same R3V6 peptide in the GBM xenograft mice model [152]. In another study by Zhou et al. modified 2′-O-methyl anti-miR were designed against miR-21 that led to successful inhibition of the expression of miR-21 in glioma cells. The downregulation of miR-21 in GBM cells induced apoptosis by activating caspase 3 and 9 [153]. Similarly, various other groups have reported the application of anti-miRs for successfully abrogating the abnormally high levels of other oncomiRs [[154], [155], [156], [157], [158], [159], [160], [161], [162], [163], [164]].

##### 4.1.2.3 miRNA sponges

Another way by which we can target oncomiRs is by designing miRNA sponges that can be used to target a whole class of related miRNAs. A miRNA sponge is designed to have multiple miRNA binding sites that act as decoys. This results in successful derepression of target genes. Chen and the group have shown that the growth of GBM can be inhibited *in-vitro* and *in-vivo* by designing a lentiviral-mediated miRNA sponge against miR-23b [165]. Since miRNA sponges can be used to target the whole family of targeted miRNA, it makes them a powerful tool against aberrant miRNA expression in cancer [166]. But their off-target effects and excessive exogenous nucleic acids in the intracellular environment raise some serious concerns associated with cellular toxicity. This is a major challenge that hampers their success as a novel therapeutic agent.

##### 4.1.2.4 Small molecule inhibitors of miRNA (SMIR)

Small molecule inhibitors are another class of miRNA targeting agents with an advantage over its predecessor antisense oligonucleotide or sponge therapy. They have high metabolic stability, greater bioavailability, and good solubility. They may target miRNAs either at the transcriptional or post-transcriptional level. Gumireddy, K et al. were the first group to identify small molecule inhibitors against miRNAs that targeted and inhibited the overexpression of miR-21 at the transcriptional level [167]. The small molecule inhibitors can also act upon specific miRNAs during their biogenesis, where they can either block the pri-miRNA processing or pre-miRNA processing by Drosha and Dicer. Bose et al. after screening multiple compounds that can potentially block the pre-miR-21 processing by Dicer, identified that the tuberculosis drug aminoglycoside streptomycin can interfere with the pre-miR-21 processing by Dicer [168]. However, the major challenge in this therapy is efficient design of these small molecule inhibitors. Till now, most SMIRs were developed by screening large libraries of pharmacologically active compounds due to the lack of efficient *in silico* tools. But, with the advent of more advanced computational and simulation tools, prediction of miRNA secondary structures has become a lot easier, paving the path for structure-based drug design.

### 4.2 Long non-coding RNAs (lncRNAs)

Long non-coding RNAs abbreviated as lncRNAs are non-protein-coding RNAs ≥200 nucleotides long which can be as long as 100 Kb in length and are highly heterogeneous transcripts [169,170]. In general, lncRNAs lack open reading frames (ORFs), but there are some that are involved in small polypeptide synthesis [169,171]. Within the cell, lncRNAs are localized in both the nucleus and cytoplasm. Inside the nucleus, lncRNAs play a key role in controlling gene expression by translocating different activation and repressor complexes and chromatin remodelling proteins to specific gene promoters. In contrast to nuclear lncRNAs, the cytoplasmic lncRNAs can control the function of their targets by trapping regulatory miRNAs and proteins [77]. LncRNAs are classified into five categories based on their archetypes, molecular functions, and mechanism of action: signal lncRNAs, decoy lncRNAs, guide lncRNAs, scaffold lncRNAs, and enhancer lncRNAs [172].

#### 4.2.1 Role of lncRNAs in GBM

Major tumor suppressor lncRNAs in GBM are RAMP2-AS1, GAS5 and CASC2 [[173], [174], [175], [176]]. Shuang Liu and his group showed that RAMP2-AS1 is downregulated in GBM and the ectopic overexpression of this lncRNA suppress tumor growth *in-vivo*. RAMP2-AS1 functions by interacting with DHC10/NOTCH3/HES1 signaling cascade and reduces GBM tumor progression by inhibiting NOTCH3 [173]. GAS5 is another tumor suppressor lncRNA, which is located on chromosome 1q25.1. The lncRNA, GAS5, interacts with the receptors of different steroid hormones by binding to their DNA-binding domains, thus preventing them from affecting the target genes [177]. In an *in-vitro* experiment, X. Zhao and his group found that GAS5 acts as a ceRNA that, when bound to oncomiR (miR-222), inhibited the growth of GBM cells [174].

Moreover, when GAS5 was overexpressed in U-87MG GBM cells, this made them sensitive to cisplatin [178]. Similar to other tumor suppressor lncRNAs, CASC2 was found to be downregulated in GBM, and its overexpression decreased the malignancy of GBM by cross-talk with miR-21 [179]. CASC2 also inhibits the Wnt/β-catenin pathway and regulates mTOR expression [176].

The major oncogenic lncRNAs involved in GBM tumorigenesis are MALAT1, NEAT1, HOTAIR, MEG3, H19 and XIST [[180], [181], [182], [183], [184], [185]]. MALAT1, also referred to as NEAT2, is one of the most abundantly expressed lncRNAs in GBM and is associated with poor prognosis in GBM patients [180]. MALAT1 is a highly conserved lncRNA and is known to regulate pre-mRNA splicing [186]. Xiang et al. revealed that MALAT1 was highly overexpressed in glioma tissue compared to other cancer types [187]. MALAT1 induces the proliferation of GBM stem cells (GSCs) by regulating the expression of SOX2 [188]. MALAT1 also aids in evading glioma cell death (apoptosis) by reducing the levels of Rap1B, a Ras-related protein, by acting upon miR-101 [189]. In a xenograft mouse model, Xiong et al. showed that MALAT1 crosstalk with miR-129 promoted glioma tumorigenesis by targeting Sox2 [188].

In GBM and other cancer types, lncRNA NEAT1 acts as a scaffold RNA interacting with multiple target genes [190,191]. The NEAT1/miR-132 axis by targeting SOX2 expression induced invasiveness in GBM cells, and thus NEAT1/miR-132/SOX2 axis could be a promising target for the treatment of GBM [190]. The study by Chen et al. demonstrated that NEAT1 by scaffolding EZH2, promotes GBM progression through Wnt/β-catenin pathway by activating miR-let-7e, which inhibited GBM stem cells invasion and migration [181]. This shows the significance of NEAT1/EGFR/EZH2/Wnt-β-catenin in the progression of GBM and could be a potential target for GBM therapy.

HOTAIR is another lncRNA that is overexpressed in GBM and other cancers located in the *HOXC* gene cluster in human chromosome 12 [192]. When Tan and his group analyzed the levels of HOTAIR in serum samples obtained from GBM patients, they found that the levels of HOTAIR were significantly higher than serum samples from the healthy group, concluding that it can be used as a biomarker for GBM prognosis [193]. Oncogenic lncRNA H19 is overexpressed in the hypoxic microenvironment, binds to its promoter under the influence of HIF-1α, thus triggering the oncogenic response in GBM [194]. LncRNA H19, by sponging miR-130a-3P, functions as a ceRNA that regulates epithelial to mesenchymal transition (EMT) of glioma cells by promoting cellular invasion and migration [195]. H19 also promotes tumorigenesis of GBM cells by recruiting EZH2 to the promoter of NKD1 [196].

#### 4.2.2 LncRNA-based target therapy to treat GBM

Since lncRNAs play a crucial role in different stages of glioma onset and progression, therefore, several novel therapeutic approaches can be considered to target both cytoplasmic and nuclear lncRNAs. This involves: 1) post-transcriptional RNA disintegration by exploiting oligo-nucleotides as therapeutics such as siRNA, antisense-oligo or synthetically altered locked nucleic acids [197,198]; 2) Transcriptional blockage of lncRNA genes promoter by using genome-editing techniques such as CRISPR-Cas9 [199,200]; 3) Steric inhibition by using small molecules such as morpholinos and altered antisense oligonucleotides to induce the loss of function of lncRNAs by blocking their interaction with targets such as miRNAs, genes or proteins [201]; and 4) Nanoparticle mediated delivery of therapeutic oligonucleotides or through inducible vectors to target lncRNAs [202]. Several studies have been reported where researchers have targeted glioma using the above approaches. For instance, the study conducted by Katsushima and his group on lncRNA TUG1 used antisense oligonucleotides delivered via drug delivery vehicles to target TUG1 in an in-vivo glioma model to repress glioma stem cells growth [203]. In another study, Kim et al. developed a targeted nanocomplex to successfully deliver siRNA against lncRNA MALAT1, effectively chemosensitizing GBM cells to TMZ [202].

The regulatory roles of lncRNAs in GBM as well as other cancers are not entirely clear. lncRNA-based anti-cancer therapy is still in the infant stage, and a better understanding of the underlying mechanism of lncRNA function is required to design effective therapeutics. Also, it remains a challenge to successfully deliver a therapeutic cargo to target lncRNAs due to the poor stability, biocompatibility, and intracellular uptake of delivery vehicles. These issues can be addressed by designing and synthesizing nanocarriers with the properties that can help them overcome the biological barriers discussed in detail later in this review.

### 4.3 Circular RNAs (circRNAs)

circRNAs are single-stranded, covalently closed-looped transcript RNA molecule that lacks a 5'end cap and 3′ end poly-A tail [[204], [205], [206]]. Earlier, circRNAs were thought to be the misspliced byproducts of introns. Only after the rapid development of transcriptomic sequencing technique (RNA-seq), circRNAs gained widespread significance, and were known to play key roles in the pathogenesis of different cancer types.

#### 4.3.1 Role of circRNAs in GBM

Similar to miRNAs and lncRNAs, circRNAs play a diverse role in GBM. Several deregulated and aberrantly expressed circRNAs in human glioma are involved in sustained proliferative signaling, apoptosis, angiogenesis, invasion, migration, chemoresistance, and radiosensitivity [[207], [208], [209], [210], [211], [212]]. The study conducted by Zhu and his group in GBM on differentially expressed circRNAs found 1411 differentially expressed circular RNAs, including 1205 down-regulated and 206 up-regulated circRNAs in GBM patients, which shows that circRNAs are highly dysregulated and aberrantly expressed in GBM and could be involved in numerous biological processes promoting tumorigenesis [213]. Several studies in human glioma have shown that circRNAs such as circ-PCMTD1, circ-PRKCI, circ-CDC45, circ-CPA4 and circ-SCAF11 are differentially expressed that are known to act as either oncogenic or tumor suppressors similar to miRNAs and lncRNA and promote glioma progression by regulating different hallmarks of cancer [[214], [215], [216], [217], [218]].

circRNAs can influence tumorigenesis in GBM in a positive or negative manner depending on whether they are oncogenic or tumor suppressors. Oncogenic circRNAs like circ-HIPK3, circ-CFH, and circ-TTBK2 act as ceRNAs by sponging miRNAs in the process of regulating carcinogenesis [[219], [220], [221], [222]]. Zhang et al. for example, demonstrated in their study that circular RNA circ- TTBK2 regulates cellular proliferation and invasion in glioma by targeting the miR-761/ITGB8 axis [222]. Bian et al. discovered that the circular RNA circ-CFH was highly overexpressed in glioma tissue and was significantly correlated with tumor grade. They also found high levels of circ-CFH in the glioma cell lines U251 and U373. They reported that knocking out circ-CFH in these two cell lines inhibited cellular proliferation by targeting the miR-149/Akt1 network [221].

On the other hand, tumor suppressor circRNAs such as circ-SMARCA5, circ-FBXW7, circ-SHPRH, and circ-PINT regulate glioma tumorigenesis by different molecular mechanisms [[223], [224], [225], [226]]. The study by Barbagallo D and his group showed that circ-SMARCA5 levels in GBM were highly downregulated and it regulates angiogenesis in GBM by targeting the SRSF1/VEGFA network. Thus, overexpressing tumor suppressor circular RNA circ-SMARCA5 can be a promising therapeutic strategy to control GBM progression by inhibiting angiogenesis [223].

#### 4.3.2 circRNAs-based target therapy to treat GBM

Several studies on circRNAs in GBM have correlated the clinical relevance of deregulated circRNAs in GBM pathophysiology. They have been reported to play roles in controlling tumor size and differentiation, GBM grading and staging, and recurrence possibility. In addition to their major functional role in regulating mRNA expression, circRNAs possess unique characteristics such as: 1) they are highly stable owing to resistance to exonucleases because of their structural variability in lack of 5′ end cap and 3’ Poly-A tail; 2) Circular RNA sequences are highly conserved across species; 3) they are widely expressed in various tissues and bodily fluids and show specificity in their expression patterns across cells, tissue and developmental origin, and 4) They are abundantly present in exosomes and cytoplasm of mammalian cells [227]. They are clinically relevant because of their presence in blood, urine, saliva, and synovial fluids [227,228]. The above properties make circRNAs ideal molecular candidates to be considered as prognostic and diagnostic biomarkers of GBM.

circRNAs such as hsa_circ_0074,027 and hsa_circ_0001649 expression has been highly correlated with progressive WHO grade tumor and large tumor size [229,230]. Whereas, certain circRNAs such as circHIPK3, circCPA4, circ_0034,642 and circ_0074,362 are associated with poor prognosis in glioma patients [215,220,231,232]. Since one of the mechanisms by which circRNAs regulate their target gene expression is by acting as a sponge to miRNAs, they can be targeted with strategies similar to targeting lncRNAs in GBM by using siRNAs and CRISPR-Cas9 engineering tools to repress circRNAs expression. Certain circRNAs such as hsa_circ_0076,248, hsa_circ_0043,949 and circ-ASAP1 are involved with TMZ resistance in GBM and can be a potential therapeutic target to restore GBM chemosensitivity to improve patient prognosis [211,233,234]. It is important to note that nanoparticle mediated therapeutic approaches to target circRNAs can be considered as a novel approach in combination with chemotherapeutics and radiotherapy to target GBM. Nanotherapeutics-based targeting impose low cytoxicity, low immunogenicity and is highly specific with less to no off-target effects in CNS.

### 4.4 Other ncRNAs known to play a role in GBM

#### 4.4.1 Piwi-interacting RNAs (piRNAs)

piRNAs are a type of small ncRNAs that have a base length of 24–35 nucleotides. They are one of the least studied ncRNAs in GBM. The piRNAs were initially known to protect the germ cells against transposons-induced genome instability by inhibiting the transcription and translation of transposons [[235], [236], [237]]. It was Aravin and his group in 2001 that first identified the role of piRNAs in *Drosophila melanogaster* gametogenesis [237]. Later, several other groups reported that piRNAs not only perform regulatory functions such as transposon silencing in germ cells but also play a significant role as gene and protein regulators in somatic cells [238,239]. In cancers, piRNAs are aberrantly expressed and play a significant role in regulating different hallmarks of cancer, such as sustained proliferative signaling, apoptosis, invasion, metastasis, angiogenesis and deregulating cellular energetics [[240], [241], [242], [243]].

In GBM tissue samples, the investigated piRNAs were mostly downregulated, suggesting their crucial role as tumor suppressors, inhibiting cell proliferation and promoting apoptosis [[244], [245], [246], [247]]. One study by Jacobs et al., in 2018 reported that pre-treatment of U87 GBM cell line with piR-8041 significantly inhibited cell proliferation and induced cell cycle arrest, which may be due to targeting the members of HSP and DNAJ family of protein chaperone and also interacting with MAPK/ERK signaling pathways. Similarly, they also found that exogenous overexpression of three other piRNAs (piR-15988, piR-20249 and piR-20249) reduced cell proliferation of U87 cells, though the degree of effect was lesser compared to transfection with piR-8041 [245]. Also, several other groups have reported the aberrant expression of piR-DQ590027, piR-DQ593109 and piR-30188 in human glioma tissues and cell lines [244,246,247]. These studies suggest that targeting the deregulated piRNAs in GBM can prove be a milestone therapy in controlling GBM tumorigenicity. However, more research is needed to fully elucidate the regulatory role of piRNAs in GBM.

#### 4.4.2 Natural antisense transcript (NATs)

These are ncRNAs which are transcribed specifically from the opposite strand of gene locus, thereby regulating expression of their sense gene [248]. NATs are located in the nucleus, and they originate from all parts of protein coding locus due to which they are highly heterogeneous [248]. NATs are known to enhance the expression of specific genes, and the high specificity to cell/tissue makes them promising agents for therapeutic applications [14]. Various kinds of RNA interference (RNAi) strategies are considered for targeting NATs such as siRNA, shRNA and antisense oligonucleotides [14]. These synthetic antisense oligonucleotides (ASOs) are known to form complex of DNA-RNA hybrid with target RNA resulting in RNase mediated RNA degradation. These ASOs have an advantage over RNAi by targeting nuclear NATs. Therefore, ASOs are also known as antagoNATS [249] for inhibiting *cis*-NATs and blocking antisense/sense transcript interaction [14]. The gene therapies can overcome practical obstacles of off-target effects, cytotoxicity or cellular uptake [250]. The NAT-targeted therapy through gene-editing has emerged as an alternative approach for therapeutics [251]. A useful gene editing tool named CRISPR-Cas9 makes a promising therapeutic strategy in battle against cancer [252]. The possibility of using CRISPR-Cas9 along with short guide RNA (sgRNA) results in knock-in/out or inhibition or activation of natural antisense RNA (NATs) in cancer. However, the limitation is that only 15 % antisense RNA can be edited by CRISPR [14]. NATs generally acts in cis rather than trans as compared to its counterparts such as miRNAs or siRNAs which acts in trans and thus due to the nuclear localization and low expression of NATs even a small change in their cis acting sites can cause a huge impact.

Also, the overlap between NATs and its counterpart gene in NAT-targeting therapy may disturb specificity and safety leading to cytotoxicity [14]. Recently, work done by Colognori et al. found a new RNA editing CRISPR tool named as “CRISPR-Csm complexes” which can be used in future to target and degrade deregulated ncRNAs. The major advantage of the Csm multi-subunit protein complex in CRISPR-Csm is that it cleaves the target RNA only in cis unlike the CRISPR-Cas complex which may induce non-specific *trans*-cleavage of cellular transcripts leading to cytotoxicity. Thus CRIPSR-Csm system overcomes the various limitations of the current therapeutic strategies targeting ncRNAs [253].

### 4.5 Drug-RNA combination therapy

Drug based RNA combination therapy have gained much focus due to tumor heterogeneity, BBB entry restriction, efflux pumps, DNA damage repair mechanism, glioma stem cells, multi-drug resistance, poor drug distribution and more as challenges faced in treating GBM. Therefore, drug-RNA strategy will not only enhance efficacy, safety, and targeted delivery by accumulating at tumor site, but can also provide synergistic effects depending on the targeted nucleic acid and chemotherapeutic drug. For example, Costa, P.M. et al., in their study targeting oncogenic microRNA-21 in GBM, reported that silencing of microRNA-21 using antisense oligonucleotides, synergistically enhanced the cytotoxic potential of sunitinib leading to decreased tumor cell proliferation [254]. The targeting of miR-21 leads to the increased transcriptional activity of repressed P53 along with the simultaneous repression of NF-kB oncogenic pathway. This could explain the synergistic effect of drug and RNA therapy when administered in combination. Similarly, a Study by Bhaskaran, V. et al., reported that targeting a microRNA cluster of 3 different miRNAs increased the cytotoxic activity of TMZ, which was not observed when the miRNAs were targeted individually [125]. The list of few of the studies involving the Drug-RNA combination study is apprehended in Table 1.

**Table 1:** List of Drug-RNA combinatorial therapy approaches tested in *in-vitro* and *in-vivo* models to treat GBM.

S. No	Drug-Nucleic acid Combination Therapy	Therapeutic Target	Model	Outcome	Reference
1)	Anti-miR-221 + TMZ	MiR-221	T98G cell line	Enhanced induction of apoptosis in TMZ resistant GBM cell line.	[255]
2)	Anti-miR-21 + TMZ	MiR-21	U-87 MG, LN229 & T98G cell line	Induction of cell cycle arrest at G2/M Phase and overexpression of miR-21 key targets (PTEN and caspase-3) in U-87MG GBM cell line.	[256]
3)	Anti-miR-21+ DOXO	MiR-21	In-vitro and In-vivo studies in LN229 cell line & Balb/c nude mice	Synergistically inhibiting the tumor growth by modulating PI3k/AKT pathway.	[257]
4)	Anti-miR-21+5-FU	MiR-21	U251 cell line	Increased apoptosis and decrease in the migratory potential of U251 tumor cells.	[258]
5)	Let-7g miRNA mimic + EPI	Let-7g	U-87 MG cell line & In-vivo mouse model	Higher transfection efficiency and significant downregulation of Pan-Ras proteins.	[259]
6)	MiR-199a mimic + TMZ	MiR-199a	U–87S cell line	Decrease in cell viability of GBM stem-like cells (U–87S) and TMZ sensitization.	[260]
7)	Anti-miR-21 + SUT	MiR-21	In-vitro studies involving U-87 MG GBM cell line & GL261 glioma cell line & In-vivo studies by developing orthotopic mouse model	Increased expression of tumor suppressor genes (*PTEN* & *PDCD4*), Caspase 3/7 activity and enhanced cytotoxic activity of SUT (Tyrosine kinase inhibitor).	[137,254]
8)	LV-miR128–3P + TMZ	MiR-128	In-vitro assays were performed in U-87 MG, T98-G, LN229, U251 & A172. Animal experiments were conducted in 6–8 weeks SCID mice	Overexpression of miR-128 by delivering LV-miR128–3P enhances the sensitivity of TMZ against GBM by modulating c-Met/EMT process.	[261]
9)	MiR-126–3P mimic + TMZ	MiR-126	GBM patients tissue samples & GBM cell lines (U-87 MG & U251)	Overexpression of downregulated miR-126–3P in GBM increases TMZ sensitization by targeting SOX2 and Wnt/β-catenin signaling pathway inactivation.	[262]
10)	pCDH-miR-140+Cathepsin B- shRNA (CTSB shRNA) + TMZ	MiR-140 & CTSB	GBM cell lines (U-87 MG & A172)	Downregulation of CTSB by miR-140 targeting, suppressed EMT transition and also sensitized GBM cells to TMZ treatment	[263]
11)	Anti-miR-20a + TMZ, and LRIG1 siRNA	MiR-20a & LRIG1	U-251 MG cell line & In-vivo xenograft tumor model	Restoration of TMZ sensitivity in GBM by downregualtion of miR-20a using miR-20a inhibitor and knock-down of *LRIG1* (target of miR-20a) using siRNA reversed the efficacy of anti-miR-20a.	[264]
12)	As-miR-21 + TMZ, SFN + TMZ & β-Catenin siRNA	MiR-21 & β-catenin	In-vitro studies in GBM cell lines (H4, SNB19, LN229, & U-251 MG) & subcutaneous GBM xenograft tumor model	SFN enhances TMZ sensitivity in GBM by decreasing the levels of miR-21 through targeting Wnt/β-catenin/TCF pathway.	[265]
13)	LV-Cas9 + TMZ	Lnc-TALC	Induced TMZ resistant cells (229R, 251R, 551WR, & HG7R) & In-vivo xenograft mouse model	Knock-down of lnc-TALC using CRISPR-Cas9 system sensitized resistant GBM cells to TMZ in both In-vitro & In-vivo xenograft mouse model.	[266]
14)	R8-PNA-a15b + SFN	Mir-15b	U-251 MG cell line	SFN and R8-PNA-a15b acts synergistically to induce apoptosis in GBM cells.	[267]
15)	R8-PNA-a221 + tetrahydrothiene [2,3-c] pyridine derivative 3b	MiR-221	U-251 MG & T98G cell line	Anti-tubulin agent (3b) & R8-PNA-a221 acts synergistically to induce apoptosis in GBM cells.	[268]
16)	Anti-miR-10b-5p + 2-(3′-chloro-4′-ethoxyphenyl)-1-(3′,4′,5′-trimethoxyphenyl)-1H-imidazole (4o)	MiR-10b	U-251 MG cell line	Anti-tubulin agent (4o) and antisense oligonucleotides acts synergistically to treat GBM by inducing apoptosis.	[269]
17)	LINC00511 ShRNA + TMZ	LINC00511	Patient samples, GBM cell lines (U-87 MG, A172, U-138 MG, U-251 MG, U-373 MG, LN‐18, & T98G) & xenograft mouse model	LINC00511 was found to be upregulated in GBM and upon silencing, it sensitized resistant GBM cells to TMZ treatment.	[270]
18)	LncSNHG15 ShRNA + miR-627–5p mimic + PALBO (CDK6 inhibitor)	LncSNHG15 & MiR-627–5p	TMZ resistant and TMZ sensitive patient derived cells & NOD/SCID mouse models	Simultaneously targeting lncSNHG15, miR-627–5p & CDK6 using shRNA + miR-627–5p mimic + PALBO suppressed tumorigenic potential of TMZ resistant cells and it further reduced the ability of TMZ resistant cells to generate M2-polarized glioma associated microglia (M2-GAM) and glioma stem cells (GSCs).	[271]
19)	E2F6siRNA + TMZ	E2F6	GBM cell lines (U-87 MG, N33, N5, N9) & orthotopic mouse model	Inhibition of *E2F6* using siRNA sensitized GBM cells to TMZ treatment.	[272]
20)	SiCDR1as + DOXO	CDR1as	U-87 MG cell line & xenograft tumor model	Overexpression of CDR1as protects P53 function when DNA damage was induced using DOXO but siRNA induced downregulation of CDR1as may promote tumorigenesis in GBM	[273]
21)	Sh-circASAP1 + TMZ	CircASAP1	GBM patients tissue, GBM cell lines & Xenograft mouse model	Overexpression of circASAP1 is highly correlated with GBM tumorigenesis and its downregulation inhibited cellular proliferation and sensitized GBM cells to TMZ treatment in both In-vitro and In-vivo tumor model.	[234]
22)	Mir-34a mimic + TMZ	Mir-34a	Primary patient derived xenograft (GBM6, GBM118 and GBM126), GBM cell lines (LN229, A172 & T98G) & orthotopic GBM mouse model	Delivery of miR-34a sensitized patient derived Xenografts and GBM cell lines to TMZ treatment. Transfection in orthotopic mouse model with miR-34a showed significant decrease in c-Met & phosphorylated Akt.	[274]
23)	Si-CRNDE + TMZ	CRNDE	Patient Tissue samples, GBM cell lines (U-251 MG & U-87 MG) & xenograft mouse model	Knockdown of lncRNA CRNDE using sensitized GBM cells to TMZ treatment and reduced the expression of a drug resistance protein ABCG2.	[275]
24)	Anti-miR-221 PNAs + anti-miR-222 PNAs + TMZ	MiR-221 & MiR-222	GBM cell lines (U-251 MG, U-373 MG and T98G)	Administration of anti-miR-221 & anti-miR-222 to target two different miRNAs simultaneously in combination to TMZ treated GBM cells had much elevated levels of pro-apoptotic effects in GBM as compared to when only one miRNA was targeted	[276]
25)	Anti-miR-155 PNAs + anti-miR-221 PNAs + TMZ	MiR-155 & Mir-221	T98G cell line	Targeting of two different oncomiRs has much higher levels of pro-apoptotic effects in GBM which was further increased upon administration of TMZ	[277]
26)	MiR-181a-5p mimics + TMZ	Mir-181a-5P	Glioma patient tissue samples and cells	Overexpression of miR-181a-5P in GBM significantly decreased the Invasiveness and migratory potential of GBM cells by targeting *FBXO11* gene. Also, Elevated levels of miR-181a-5P in GBM cells increased their chemosensitivity to TMZ treatment	[278]

**TMZ**: temozolomide; **DOXO**: **doxorubicin; FU**: fluorouracil; **EPI**: epirubicin; **SUT**: sunitinib; **SFN**: sulforaphane; **PALBO**: palbociclib**.**

## 5 Challenges for effective RNA-based molecular therapy

The two major challenges in RNA therapeutics are overcoming the anatomical barriers and to deliver RNA-based cargo directly to the target site shielding it from degradation. Nucleases are present abundantly in the plasma or intracellular environment system and the need to deliver RNA payload without degradation is important prior reaching to the requisite site. Various nanocarriers have been designed for effective delivery of RNA-based therapeutics in GBM. These delivery agents must be of small size preferably in the nanoscale range, be able to evade immune response, facilitate cellular uptake, cause low cytotoxicity, and protect the cargo from nucleases [279]. Inorganic nanocarriers (gold, silica, iron oxide, silver) could be synthesized for attaining controlled ultrasmall size for overcoming anatomical barriers. While organic nanocarriers (lipid-based or polymeric) have made progress in size controlling but somehow still lack tunability as well as stability compared to inorganic or metallic nanoparticles. Scale-up at bulk level is also challenging for organic nanocarriers. The main focus of RNA-based therapeutics remains potentially targeting and silencing off disease causing genes. In 2018, ‘Patisiran’; a lipidbased nanoformulation became the first FDA approved delivery system for RNAi therapeutics [280]. They further can be conjugated with targeting moieties or surface modifications can be carried out which can potentially lead to specific targeting with less immunogenicity as well as toxicity. In the section we outline the various challenges associated with the RNA therapeutics.

### 5.1 Delivery challenges to treat GBM

#### 5.1.1 Blood-Brain Barrier (BBB)

**Fig. 3:**
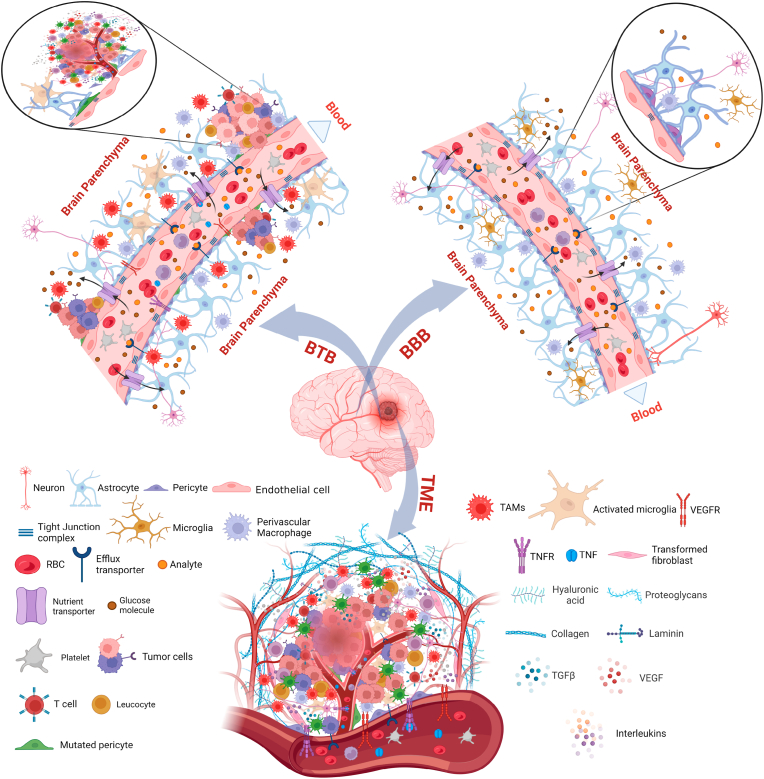
Major challenges to treat GBM. Blood-brain barrier (BBB), blood tumor barrier (BTB) & Tumor microenvironment (TME) are the 3 major challenges while designing effective therapeutic strategies to treat GBM. The figure also illustrates the structure of normal BBB and compares the BBB with the BTB apart from showing the cellular and vesicular nature of the TME in GBM. Some of the most prominent difference in BTB from BBB is immune cell infiltration which includes but is not limited to TAMs, activated microglia and T-cells etc, cancer cell migration, colonization and the neuroinflammatory response. Significant changes include the detachment, translocalization and loss of astrocyte endfeet processes by nearby invading tumor cells, aberrations in pericytes, increased VEGFR and TNFR expression on the surface of endothelial cells and disruption of tight junction complexes. Figure created with BioRender.com

**Fig. 4:**
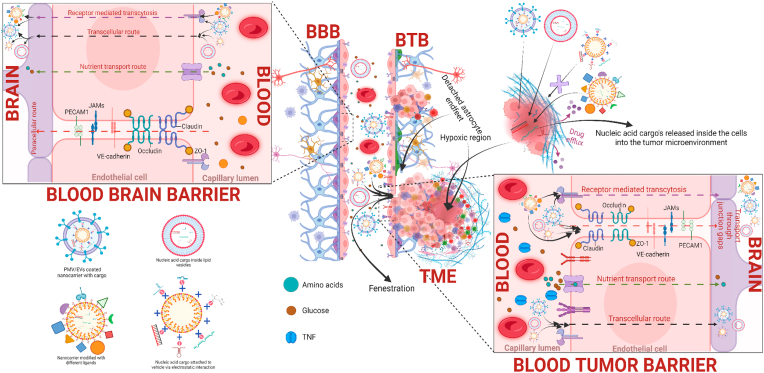
Transport mechanism of nucleic acid cargo through various routes using nanocarriers with different modification strategies to overcome challenges associated with GBM for effective therapy. Different types of nanoparticles encapsulating nucleic acid cargo can follow different routes of transport across BBB, BTB and TME depending upon the parameters such as; 1) Physiochemical. Figure created with BioRender.com

The BBB acts a critical and primary factor for the successful design and treatment of many CNS diseases. BBB is considered as the toughest anatomical barrier in the biological system which protects and regulates hemostasis in the brain [281]. It is mainly composed of endothelial cells lining the inner surface of blood vessels. These endothelial cells are bound tightly together through tight junctions, and are covered by a special basal lamina along with pericytes and astrocytes interconnecting neural endings (Fig. 3, Fig. 4) [282,283]. The high specificity and selective nature of BBB corresponds to 98 % restricted entry of large and small molecules, Although the barrier is compromised and has a leaky vasculature in GBM patients still, the rate of entry is limited in the case of therapeutics for the treatment of GBM and other neurological diseases [17,284,285]. This leads to inhibition in transport of systematically administered therapeutic drugs, polar macromolecules or antibody tagged vaccines. Through transcellular route, BBB allows small hydrophobic lipid materials and passage is generally receptor mediated (Fig. 4) [286]. Therefore, the transport across BBB depends upon either molecular weight of the carrier or lipophilicity through passive mechanism or receptor mediated (Fig. 4) [287,288]. Thus, the engineered smart nanomaterials modified with BBB-penetrating ligands for tumor targeting have shown promising results for treating CNS tumors.

Efflux pumps in BBB is known to restrict entry of harmful chemicals into the CNS. Proteins of the ATP binding cassette (ABC) transporter family are being recognized as key regulators of drug transport across the BBB. P-glycoprotein (P-gp) is a major efflux transporter that restricts the entry of lipophilic drugs. Apart from P-gp, the BBB is also reinforced by breast cancer resistance protein (BCRP) [289]. These drug efflux pumps act as an extra line of barrier across BBB. Due to the efflux pumps, innumerable strategies in clinical trials have failed which started with the assumption that the therapeutic suboptimal agent is able to surpass BBB. Designing of PI3K/mTOR pathway inhibitors having less affinity to efflux transporters was found to be more efficient due to better penetration and molecular targeting [290,291]. In a series of experimental demonstration by Becker et al. efflux transporters remain important for maintaining the integrity of BBB and blocking the drug penetration. A better understanding of the molecular mechanisms and signaling cascades responsible for drug efflux is an absolute requirement before moving on to clinical trials [292].

#### 5.1.2 Blood Tumor Barrier (BTB)

In GBM, the typical BBB architecture gets disrupted as the tumor progresses and is thereafter referred to as the blood-tumor barrier (BTB) or blood-brain tumor barrier (BBTB). BTB is yet another major obstacle that has hampered the development of effective treatment strategies to treat GBM for decades [293,294]. With the progression in GBM neovasculature, the BBB is transformed to BTB surrounding the tumor, becoming leakier with subtle changes in its physiological structure. BTB cannot be defined as a separate structural entity as it occurs within the tumor and the blood vessels surrounding it [283]. Its structural integrity is governed by factors such as immune cell infiltration, cancer cell migration, colonization and the neuroinflammatory response (Fig. 3) [283]. Significant changes that occur during the transition of BBB to BTB include: 1) the detachment, translocalization and loss of astrocyte endfeet processes by nearby invading tumor cells; 2) aberrations in pericytes, disrupting the BBB integrity; 3) increased VEGFR and TNFR expression on the surface of endothelial cells and; 4) disruption of tight junction complexes leading to increased permeability through paracellular route (Fig. 3) [283,295]. Although there is an increased permeation of molecules across the BTB, still to date, the role of BTB is still debatable and is not fully understood due to its structural complexity, tumor heterogeneity and immune cell invasion [295,296]. Therefore, its impact on the therapeutic outcome cannot be completely elucidated. Thus, smart strategies need to be designed such as engineered nanocarriers that can effectively overcome the transformed BBB to treat GBM.

#### 5.1.3 GBM tissue microenvironment

The extracellular microenvironment next to tumor is referred to as tumor microenvironment (TME). GBM is known to have an acidified extracellular tumor microenvironment similar to any other type of solid cancer [297]. The extracellular acidic environment is known to favor tumorigenesis and further escalates the tumor growth at decreased pH levels leading to increased proliferation, resistance to apoptosis, tumor cell invasion, enhanced angiogenesis and resistance to autophagy, thereby promoting multi-drug resistance and therapeutic failure of studies in clinical trials [298,299]. There are four zones described corresponding to GBM microenvironment depending upon distinct cellular phenotypic nature and dysregulated pH levels. The TME includes “necrotic zone” constituting pH levels ≤3.4, near-by surrounding with pH levels of < 5.5 referred to as “pseudo-palisading cell zone”, the “cellular tumor zone” exhibiting pH in the range of 6.2–7.0 and lastly with normal pH values referred as “leading edge zone” [300]. Among these, the necrotic core and the pseudopalisdes are highly hypoxic in nature. The lack of nutrients, and growth factors induce the expression of pro-angiogenic factors such as vascular endothelial growth factor A (VEGFA), which promotes angiogenesis. The GBM TME consists of various types of cells such as astrocytes, neurons, microglial cells, tumor associated macrophages (TAMs), dendritic cells (DCs), neutrophils, pericytes, and endothelial cells. Cytokines released by the tumor cells, TAMs, and microglial cells render the GBM TME immunosuppressed, further promoting immune evasion. Apart from these, the GBM TME has been reported to harbor cancer stem cells (CSCs), owing to the hypoxic nature of the TME (Fig. 3) [[301], [302], [303]]. Overall, the GBM TME appears as a major hindrance towards effective therapy. The obstacle of highly acidic TME could be reduced with the use and delivery of de-acidifying agents. In a report, bafilomycin A1 and chloroquine incorporated inside silica nanoparticles demonstrated accumulation in endo-lysosomes as de-acidifying agents [304,305].

## 6 Targeted therapy for GBM

The current modes of GBM therapy primarily include surgery followed by chemo and radiotherapy. However, complete surgical removal of the tumor is almost impossible, and chemo/radiotherapy bears risk of toxicity towards non-tumor healthy cells. As a result, targeted therapy is gaining more importance where the drug or therapeutic agent is securely delivered to the tumor site without affecting the nearby healthy cells. Thus, off-target effects can be avoided. Even for miRNA or other ncRNA-based therapy, a targeted mode of approach is preferable. Nanoparticles have proven to be especially useful for targeted delivery of RNA-based therapeutics to GBM cells. Targeted therapy to treat GBM can be either active or passive depending upon the mode of therapeutic delivery and moieties attached.

### 6.1 Passive targeting

Passive targeting involves the delivery of anti-cancer therapeutics specifically to tumor site using distinct inherent properties of the cancer cells. This effect, first identified by Matsumura, and Maeda in the year 1986, is known as Enhanced permeability and retention (EPR) [306]. The EPR effect highlights the leaky, and dilated vasculature at the core of solid tumors that arises from abnormal angiogenic signaling. These blood vessels allow nanoparticles/macromolecules to extravasate into the tumor tissue, while normal blood vessels having intact gap junctions do not [306]. Many groups have exploited this unique property of GBM (solid) tumors to deliver chemotherapeutic drugs or RNA-based therapeutics to tumor site. GBM tends to have a permeable tissue vasculature due to hypervascularization, and hypervascularization leads to physical disruption of robust endothelial BBB junction [307,308]. This pathophysiology affects BBB integrity which allows moieties or nanocarriers to penetrate BBB. Another important factor that plays a role in passive transport is the interplay between the hypervascularization and hypoxia in GBM. The hypoxia driven tumor cells overexpress factors such as hypoxia inducible factor-1α (HIF-1α) and vascular endothelial growth factor (VEGF) which promotes hypervascularization in GBM and thus this endless cycle continues. In a study on mouse mammary model by Chauhan et al. showed that by reducing tumor vascularization through VEGF receptor blocking, nanoparticles could be delivered more efficiently in a size dependent manner [309]. Though the study was not in direct context to GBM but it gives insight into developing effective targeting strategy by targeting tumor hypervascularization and that such strategies could be employed to passively target GBM. Similarly, in another study, adenosine receptor agonist (A2A) named lexiscan which is an FDA approved agent for myocardial perfusion imaging has been used in orthotopic glioma mouse model. The integration of lexiscan increased the permeation of nanoparticles across BBB with enhanced therapeutic payload efficacy [310].

In another study, liposomal paclitaxel was delivered to mouse brain tissue through focused ultrasound allowing 2-fold higher drug concentration delivery and better survival in intracranial GBM derived mouse model [311]. Seo JW in 2015, studied the effect of EPR as a function of nanoparticle size. After systematic administration of Cu labelled nanoparticles in intracranial rat GBM model. They tracked through MRI and PET after 7 h of administration; the uptake of 20 nm nanoparticles was higher than 110 nm nanoparticle [312]. However, the passive targeting of GBM has not been very successful as the drug might accumulate at the neoplastic tissues due to the EPR effect, but their uptake by the cancer cells is not facilitated by this method. The challenges in passive targeting mediated by EPR effect can be addressed by active targeting.

### 6.2 Active targeting

As discussed earlier, passive targeting may promote the localization of the drugs/therapeutics to the tumor site but cannot facilitate their cellular uptake. This problem can be resolved by active targeting method where the drug or payload is specifically delivered to the cancer cells or even subcellular locations, using nanoparticles decorated with targeting ligands that are specifically recognized by cancer cells. The receptor mediated active targeting of GBM can be achieved by modifying the nanocarriers surface with ligands such as insulin, transferrin, lactoferrin, amino acids and angiopep-2 etc. [[313], [314], [315], [316], [317], [318], [319]]. Other than this, active targeting of GBM using nanocarriers can also be achieved by modifying them with either peptides such as iRGD, chlorotoxin, T7 (HAIYPRH- binds transferrin receptor) and F3 (KDEPQRRSARLSAKPAPPKPEPKPKKAPAKK- binds nucleolin) or using small molecules such as folic acid or it can also be accomplished by cytokine mediated targeting of GBM using Interleukin-I3 specific to IL-I3Rα2 receptor [[320], [321], [322], [323], [324]].

Nanoparticles are mainly surface modified depending upon specificity to BBB or glioma. There are various types of modifications applied on polymeric nanocarriers depending upon the specificity to the target or their application. For instance, nanoparticles of poly butyl-cyanoacrylate loaded with doxorubicin and surface coated with P80 as surfactant showed the increase in survival rate of GBM bearing rats compared to those treated with the non-coated ones [325]. Similarly, study performed by L Kong and colleagues in 2016 showed efficient delivery of (arginine-glycine-aspartic) a functionalized dendrimer-entrapped gold nanoparticles for siRNA delivery into GBM cells by inducing gene silencing *in-vitro* as well as *in-vivo* [326]. Also, Cohen et al. synthesized and delivered hyaluronan conjugated lipid-based nanoparticles for RNAi therapy against chemo resistant grade IV GBM [327]. Similarly, Yu et al. showed RNAi therapy for GBM using lipopolymeric nanoparticles delaying its progression [328]. Shah N. et al., in 2009 showed PLGA [poly (lactic-co-glycolic acid)] nanoparticles loaded with paclitaxel conjugated with transferrin (BBB ligand) were readily taken up by C6 rat glioma cell line supporting receptor mediated endocytosis and reduced toxicity [329]. Miura Y. et al., in 2013 reported the targeted efficacy of cRGD (Arg-Gly-Asp), grafted on polymeric micelles showing enhanced uptake of micelles by orthotopic mouse GBM model. The cRGD ligand showed selectivity for integrins ɑvβ3 and ɑvβ5 which are overexpressed in tumor cells and the RGD micelles encapsulated with oxiplatin reduced tumor progression [330].

The liposomes preserve versatility with advantage of encapsulating hydrophilic drugs in aqueous compartment whereas hydrophobic drugs in bilayer. Therefore, PEGylating nanoparticles may neutralize the surface charge and thus enhance circulation of the nanoparticles in the biological system. Liposomal formulation containing doxorubicin + PEGylated (Caelyx®) showed higher accumulation of doxorubicin in GBM patients intratumorally than normal brain tissues [331]. The positively charged liposomes show adsorption mediated endocytosis through electrostatic interactions to pass through the BBB [332]. Liposomes encapsulated with 5-fluorouracil and conjugated with Transferrin had enhanced brain delivery permeating BBB 13 times higher as compared to non-conjugated [333]. Also, liposomes grafted with interleukin-13 enhanced cytotoxicity and tumor accumulation of doxorubicin than free drug subcutaneously delivered in mouse glioma model [334]. Therefore, liposomes or lipid nanocarriers surface attachment of HS15 surfactant suppressed the efflux pump P-gp, ensuring efficient brain targeted delivery [[335], [336], [337]].

Reports suggest that metallic or magnetic inorganic-organic nanoparticles can be used for diagnostic as well as therapeutic purposes. PLGA + paclitaxel loaded conjugated superparamagnetic iron oxide nanoparticles increased median survival rate than passive targeting in U87 MG orthotopic mouse tumor model [338]. In a study in 2019 reported by Zhao L. et al., ^131^I-labelled chlorotoxin peptide (CTX-specificity in blocking Cl channel in glioma) functionalized polyethyleneimine entrapped gold nanoparticles for glioma radionucleotide therapy with SPECT/CT imaging; (Single photon emission computed tomography). The C6 cells showed enhanced cellular uptake with higher fluorescent emission intensity observed under confocal fluorescence spectroscopy. These nanoparticles can also be used as nanoprobes owing to their multifunctional glioma-targeted properties [339]. Nevertheless, the delivery of therapeutic payload using nanocarriers for efficient delivery via passive or active targeting has great potential for translating from the bench to the bedside. The list of few of the studies with receptor-ligand mediated active targeting to treat GBM is apprehended in Table 2.

**Table 2:** Receptor-ligand mediated active targeted delivery of therapeutics in *in-vitro* and *in-vivo* studies for GBM therapy.

Target Receptor	Ligand	Ligand Modified Vectors	Therapeutics Delivered	Study Type	Reference
TfR	Cyclic targeting peptide (CRTIGPSVC), transferrin, T7 peptide and lactoferrin	Viral-phage clone, Fused VEGF-TfR targeting bispecific antibodies, PEGylated liposomes, mesoporous ruthenium nanoparticles, exosome-mimetic cell membrane vesicles, lactoferrin nanoparticles, mesoporous silica nanoparticles, liposomes, PLGA and co-micelles prepared from cholesterol conjugated T7 peptide etc.	HSV thymidine kinase gene, VEGF antibody, resveratrol, ruthenium complex (RBT), siRNA, TMZ, bortezomib, docetaxel and anti-miR-21	In-vitro, orthotopic and subcuteaneous mouse and rat model.	[[340], [341], [342], [343], [344], [345], [346], [347], [348], [349]]
IR	Human IR specific mouse monoclonal antibody (MAb), murine 83-14 MAb and rat 8D3 MAb	PEGylated immunoliposomes,	Human EGFR antisense gene, EGFR-shRNA	In-vitro & orthotopic xenograft GBM mouse model	[350,351]
IL-13Rα2	Mutated IL13, recombinant cytotoxin IL13-PE38QQR and human interleukin-13	Chimeric plasmid “pLenti-IL13 (4MS)-EphA2 scFv-TanCAR”, plasmid with T7 bacteriophage promoter, human IL-13 modified liposomes, pulsed dendritic cells,	Bi-specific antibody IL13 (4MS)-EphA2 scFv-TanCAR backbone, recombinant chimeric cytotoxin, doxorubicin, CTL epitope peptides IL-13Rα2_345–353_ (WPFGFILI) and IL-13Rα2_174–182_ (WYEGLDHAL),	In-vitro, subcuteaneous xenograft GBM mouse model, phase III clinical trial, phase I clinical trial	[[352], [353], [354]]
FR	Folic acid	polyhedral oligomeric silsesquioxane nanoparticles, PLGA nanoparticles, biopolymeric nanoparticles, hollow titanium dioxide nanospheres, tri-block copolymer nanoparticles, cationic lipoplexes	TMZ, curcumin, doxorubicin, gefitinib, eGFP-pDNA	In-vitro, orthotopic and subcuteaneous mouse model	[[355], [356], [357], [358], [359]]
ITR	iRGD peptide and cRGD peptide	Liposomes, H-ferritin nanocarrier, PEG–PTMC nanoparticle, solid-lipid nanoparticles, PLGA nanoparticles	Docetaxel, doxorubicin, Paclitaxel, siRNA, anti-miR-21 and anti-miR-10b	In-vitro & orthotopic and subcuteaneous xenograft GBM mouse model	[349,[360], [361], [362], [363]]
nAChR	RVG29 peptide, CDX (cgreirtgraerwsekf, D-form sequence) peptide	PEG-PLGA nanoparticles, zein based nanoparticles, dextran nanoparticles, albumin nanoparticles	Docetaxel, dactolisib, paclitaxel, TGF-β receptor I (TGFβRI) inhibitor (LY2157299), mTOR inhibitor (celastrol)	In-vitro, orthotopic GBM rat and mouse model	[[364], [365], [366], [367]]
LRP-1	angiopep-2 peptide and RAP12 (EAKIEKHNHYQK)	Exosome conjugated magnetic nanoaprticles, solid-lipid nanoparticles, pH-sensitive polymersomes, PEG–PLA micelles, virus-mimicking polymersomes	siRNA, brequinar (BQR), docetaxel, doxorubicin, paclitaxel, chaperone saporin (SAP)	In-vitro, orthotopic xenograft GBM mouse model	[[368], [369], [370], [371], [372]]
GLUT1	GLUT1 antibody single chain fragment variable (scFv) and ginsenoside Rg3	Micelles, liposomes	Doxorubicin, curcumin, paclitaxel	In-vitro, orthotopic xenograft GBM mouse model	[373,374]
MMPs	MT1-AF7p peptide (HWKHLHNTKTFL) and chlorotoxin (CTX)	PEG–PLA nanoparticles, PLGA nanoparticles	Paclitaxel, morusin	In-vitro, orthotopic xenograft GBM mouse model	[375,376]

**TfR:** transferrin family receptor; **IR**: insulin receptor; **IL-13Rα2**: interleukin-13 receptor subunit alpha-2; **FR**: folate receptor; **ITR**: integrin receptor; **nAChR**: nicotinic acetylcholine receptor; **LRP-1**: lipoprotein receptor protein 1; **GLUT1**: glucose transporter-1; **MMPs**: matrix metalloproteinases.

## 7 RNA delivery systems for GBM

The advancement in our understanding of cancer molecular biology and evolution of gene therapy has set a new direction for GBM treatment [377] (see Table 3). The possibility includes activation or inactivation of various targeted tumor suppressors/oncogenes and growth factors or their receptors through DNA/RNA delivery systems [378]. These targeted therapeutic delivery systems carry smart macromolecules which are being investigated at pre-clinical or clinical stages. RNAi along with gene therapy has shown promising results and has reached up to the first clinical trial stage [379,380]. Broadly, RNA delivery systems are characterized into two categories; a) Viral vectors b) Non-viral vectors.

### 7.1 Viral vectors

Delivering RNA-based therapeutics using virus as delivery vehicles is an interesting approach as they can inject their genetic material into host cells, and increase copy number when integrated into DNA/nucleus of host cell [381]. The advantage of using virus as vector for delivering therapeutic agent (compound of interest) is its structural ability to escape enzymatic degradation. There are numerous kinds of viruses used in gene therapy as delivery vehicles namely; adenoviruses, retroviruses, herpes simplex viruses and adeno-associated viruses [382]. The viral vectors have been developed as targeted gene delivery systems to directly transcribe for infectious RNA transcripts [381]. Gene delivery systems based on viruses mainly includes onco-retro viral vectors, adenoviruses, adeno-associated viral vectors & lenti-viral vectors [[382], [383], [384]]. For example: Luan et al., in 2015 delivered miR-100 mimics using lenti-viral vector to overexpress the highly downregulated miR-100 in GBM [385]. The viral vector system for the delivery of nucleic acids holds advantages in terms of higher transfection efficiency and delivery of large DNA molecules but they fail as they elicit a strong immune response [386,387]. Therefore, the use of non-viral vectors such as different classes of nanomaterials has emerged as a promising tool for the efficient delivery of nucleic acid cargo to the target tissues.

### 7.2 Oncolytic viral vector therapy

One of the emerging delivery vehicles for targeted treatment against cancer type diseases are oncolytic viruses integrated vectors (OVs) [388]. OVs are biological cocktails of engineered viruses that are delivered to tumors via intravenous, or intratumoral routes and cause direct oncolysis by immunosensitization. In 1991, Ezzeddine et al.*,* deployed gene edited herpes simplex virus (HSV) to selectively kill glioma cells [389]. In another report oncolytic adenovirus expressing interleukin (IL-12 and IL-18) were found in improving tumor specific immunity in differentiating T-cells, which expressed IL-12Rβ_2_and IL-18Rα [390]. Another group has shown that adenovirus mediated decorin induces cancer cell death and mitochondrial apoptosis by activating p53 [391]. Although OVs have great potential, their major limitation is that they are prone to elicit strong immune response. As a result, most of the OVs are cleared from the system and only a small amount of the initial payload is delivered to the tumor site. More safe vectors need to be designed for an efficient therapy.

### 7.3 Non-viral vectors

Viral vectors are risky as they may potentially elicit adverse immune reaction, even after disarming the virus. Non-viral vectors on the other hand are less likely to induce immune response as biocompatible materials such as lipids, polymers, inorganic/metal organic frameworks, nucleic acid or proteins and conjugate complex delivery systems are used as vehicles [[392], [393], [394]]. They are simpler in theory and complex in applicability. Since there is no-fit-in-all theory, so numerous new compounds, formulations, new developments, various nanocarriers etc. are all continuously proposed and investigated. Every day, improved strategies and advancements involving non-viral vectors are employed for efficient delivery of RNA-based therapeutics [392].

The non-viral nanocarriers are proposed for introducing genetic material artificially through physical methods into cancer cells [395]. Recent preclinical as well as clinical data proves nanocarrier mediated delivery in/or combination play pivotal role for attaining efficacy in terms of therapeutics by overcoming tumor heterogeneity, multi-drug resistance or dose-limiting toxicity [381,395]. Also, as per previous reports in the recent years, nanocarriers have attained much attention for possibly overcoming unfavorable hurdles, majorly crossing the BBB [394]. The BBB restricts 100 % of larger molecules and 98 % of smaller molecules from entering the brain and achieving relevant therapeutic dosage. Therefore, nanocarriers encapsulating RNA-based payload can be tailored for specific transport to brain or crossing of anatomical barriers enabling them to reach the target site in difficult to reach tumors such as GBM [393,396].

An ideal RNA/chemodrug nanocarrier should have high encapsulation efficiency, should be able to protect the cargo from RNase mediated degradation, displacement by other polyanion moieties, enhance its half-life, and also ensure a sustained release [397]. For example, the half-life of the chemotherapeutic drug TMZ was increased to 13 h as compared to 1.8 h after encapsulating it inside chitosan-based nanocarrier [398]. The use of nanoparticle-based treatment strategy against various cancers has been approved by FDA almost two decades ago such as polymeric carrier PLGA or inorganic silica for human consumption. However, nanocarrier mediated studies are still ongoing with increasing number listed in clinical trials in case of GBM. The major delivery systems developed till date include inorganic (metal or metal oxide nanoparticles, silica-based nanoparticles) or polymer-based (natural or synthetic), lipid-based (liposomes, solid lipid nanoparticles, transferosomes, micelles) and carbon-based (fullerenes, carbon nanotubes, graphene's) agents for drug/RNA delivery to brain [255,[399], [400], [401], [402]]. Apart from these, in recent years there has been a huge advancement in the macromolecule-based drug delivery vehicles such as DNA/RNA or proteins and extracellular vesicles such as exosomes due to their unique properties such as specificity, tissue homing, innate stability, structure predictability and programmability [[403], [404], [405]].

characteristics of a nanoparticle such as size, shape and charge, 2) chemical composition of the entities used for the synthesis of nanoparticles, 3) surface modification of a nanoparticles for active targeting to GBM which involves modification with different ligands onto nanoparticle surface, 4) Surface coating of a nanoparticle for active targeting to GBM tumor microenvironment by homotypic recognition etc. For example, in BBB, nanoparticles modified with ligands are endocytosed by receptor mediated transcytosis. Similarly, plasma membrane vesicle (PMV) coated nanoparticles are uptaken by either receptor mediated transcytosis or by transcellular route [295]. Whereas the transport of nanoparticles across BTB can also happen by enhanced permeation and retention (EPR) effect through junction gaps between two endothelial cells [283,406]. The therapeutic cargo delivery strategies generally fail to deliver cargos to the tumor microenvironment if they are not modified with receptor specific to cancer cell types or shielded with cell membranes due to dysregulated pH or because of the presence of immune cells rich environment [407]. For cell types, details of receptor and ligands and small molecule across BBB, BTB and TME, please refer to figure (3).

#### 7.3.1 Lipid-based nanocarriers

The potential capabilities of lipid nanocarriers as gene delivery systems have been evaluated for cationic lipids constituting of liposomes, lipid nano-emulsions or solid lipid nanoparticles. Positively charged hydrophilic head (polar) binds via electrostatic interaction with negatively charged phosphate group of nucleic acid i.e., hydrophobic tail (non-polar symmetric or dissymmetric) at physiological pH leading to the formation of lipoplexes. This cationic lipid/nucleic acid entity i.e., lipoplex plays a vital role in delivering them inside cells by interacting with the negatively charged cell membrane. The lipoplexes escape the endosomal pathway, where DNA dissociates from cationic lipid and moves through cytoplasm into nucleus uncoated [392,394,408]. Lipoplexes are partial condensed DNA complex with irregular morphology. Lipoplexes as delivery systems have shown promising role as non-viral delivery carriers and few have also made to clinical trials [408,409].

##### 7.3.1.1 Liposomes

Liposomes have been used from ages as delivery vehicles to deliver drugs or genes as they are a) cheap and non-toxic, b) offer overall protection against enzymatic degradation, c) can deliver large portion of biological component (DNA/RNA), and moreover d) can attain targeted delivery. These spherical in shape vesicles made up of phospholipids can range in size from 20 nm to microns. These carry forward the advantage of instant complex formation when cationic liposomes (positively charged group) interact with DNA/RNA (negatively charged) possessing 100 % encapsulation efficiency. For having efficient delivery with liposomes, ratio of lipid to DNA must be considered carefully while forming lipid complexes [409,410]. The major drawback of liposomes is their short half-life in the systemic circulation, systemic toxicity particularly when using cationic lipids and stability which can be overcome by varying the kind of a lipid used, their composition and surface modification.

In reports, several lipid-based nanocarriers have been employed for delivering nucleic acids (DNA or RNA) to tumor, epithelial cells, endothelial cells, hepatocytes etc [[411], [412], [413], [414]]. These can be delivered through various routes of administration such as intravenously or intratissue into animals. In a case study, transfection of NIHT3 cells with lipofectamine along with DNA labelled with rhodamine, resulted in nuclear uptake of the cargo within 0.5–1 h of treatment clearly visualizing the presence of naked DNA (delivered as cargo) into nucleus crossing nuclear membrane by passive diffusion [415].

##### 7.3.1.2 Lipid nano emulsions

Nano emulsions are thermodynamically stable isotropic system which includes dispersion of 2 immiscible solution phases (water or oil) mixed and stabilized by an emulsifying agent to form a single phase. The method includes oil, water or surfactant emulsions. The dispersed emulsion mainly comprises of particles ranging up to 200 nm and is considered to be advantageous than liposomes having superior properties as scaling up (can be produced on industrial scale), high stability (during storage), and serum resistant with overall less toxicity (biocompatible). These include physiological lipids as drug vehicles such as cholesterol, esters, phospholipids and triglycerides. Moreover, the addition of cationic lipids as surfactant makes them ideal and suitable as delivery carriers [416]. These cationic lipid surfactants allow positively charged droplets formation with strong electrostatic interactions between nucleic acid (anionic) phosphate groups with emulsion (cationic). Few examples of lipid nano emulsions include DOTMA (1,2-di-O-octadecenyl-3-trimethylammonium propane), DOTAP (1,2-Dioleoyl-3-trimethylammonium propane), DLin-DMA (1,2-dilinoleyloxy-3dimethylaminopropane) and DC-Chol. In a case study, Bruxel et al. formulated cationic nano emulsion along with dotap encapsulated oligonucleotides targeting malarial topoisomerase II as delivery systems [417]. Cationic nano-emulsion has shown efficacy as delivery systems with higher transfection rate and low toxicity as compared to liposomes. Also, reports containing cationic amino-based nano emulsion have been reported in literature. For further enhancement of stability and non-aggregating ability due to steric hindrance, nonionic surfactant such as PEG could be incorporated. This strategy will also overcome enzymatic degradation and opsonization process, resulting in prolonged circulation in blood [418,419].

##### 7.3.1.3 Solid lipid nanoparticles (SLNs)

These are developed by high pressure homogenization technique without using organic solvents and contain both properties of liposomes and lipid nano-emulsions. They remain solid at room temperature or body temperature. Therefore, by combining advantages of colloidal systems they also exhibit physiological properties of polymeric nanoparticles such as sustained drug release and can be modulated accordingly. These nanoparticles have shown good stability even in lyophilized form facilitating scale-up at industrial level. SLNs consisting of at least one cationic lipid have been used as suitable non-viral vectors for nucleic acid delivery. They also exhibit nucleic acid protection against nuclease degradation. For example; SLNs resist DNase I mediated degradation of nucleic acids by binding to them and delivering them to target site [420]. During electrostatic interactions, SLNs enhance transfection by condensation as it facilitates the mobility and prevents DNase and RNase mediated degradation of nucleic acids as cationic character generates enough positive charge on the surface. For successfully protecting the nucleic acid cargo from degradation by nucleases, and enhancing transfection efficiency, an optimum (w/w) ratio of the nucleic acids and SLNs needs to be achieved. In a case study, SLN was used as a delivery agent for gene therapy against ocular diseases. After administering SLN vector carrying EGFP to mice eyes, EGFP expression was notable [421]. However, the response also depends upon the route of administration such as intravitreal or retinal. In another *in-vivo* study, protein expression was detected when SLN incorporated with EGFP plasmid was administered intravenously in BALB/c mice. The response observed in the liver and spleen remained for at least 1 week from 3rd week of administration. Further, when dextran and protamine were incorporated inside SLNs, transfection efficacy improvement was recorded *in vivo* [422].

##### 7.3.1.4 RNA-based therapeutics delivered using lipid nanocarriers in GBM

The use of nanoparticles for efficient delivery and controlled release of oligonucleotide therapeutics to tumor site can reduce high therapeutic dosage requirement, resulting in minimum systemic and cellular toxicity [423]In a study by Shabana M. et al., complexation of miR-603 with PEI (polyethyleneimine) encapsulated in liposomes enhanced the radiation sensitivity of GBM stem like cells derived from patients by downregulating the IGF1 (insulin-like growth factor 1) signaling. They used a targeted delivery approach by decorating the surface of the liposomes using PEG and PR_b which is a peptide that mimics fibronectin and targets the α5β1 integrin overexpressed on the surface of GBM cells [424]. This resulted in an increase in the intracellular miR-603 levels by 22-fold which led to downregulation of IGF1 and IGF1R (receptor) transcript levels. In another study, Costa M. Pedro et al. demonstrated the targeted delivery of anti-miR-21 oligonucleotides to an orthotopic mouse model of GBM using chlorotoxin (CTX) coated stable nucleic acid lipid particles (SNALPs) (Table 3). The peptide CTX is a well-known marker for glioma that has been derived from scorpion. The authors have shown considerable accumulation (8.3 ± 2.2) fold change increase of SNALP-FAM-oligonucleotides fluorescence at the tumor site with minimum off-target effects when administered systematically compared to non-targeted (NT)-SNALPs (5.6 ± 1.7, p < 0.05). Also, no significant differences were observed in normal brain for CTX-coupled SNALPs compared to NT-SNALPs and simple saline. Successful downregulation of the oncomiR led to an increase in the transcript and protein levels of RhoB, decreasing tumor progression. The intravenous administration in GBM bearing mice showed enhanced apoptosis activation along with improved survival rate (>30 days) when anti-miR-21 therapy was coupled with the tyrosine kinase inhibitor sunitinib. Thus, SNALPs offer a reliable and efficient delivery system for anti-miRNA nucleotides for GBM therapy [137].

**Table 3:** Therapeutic efficacy of nanoparticle mediated nucleic acid cargo delivered to treat GBM in *in-vitro* and *in-vivo* studies.

Nanoparticle	Nucleic acid cargo	Targeting moiety	*In-vitro* assessment	*In-vitro* efficacy	*In-vivo* assessment	*In-vivo* efficacy	References
Liposomes	miR-603	PR_b	Radiation sensitivity	3 fold increase in radiation sensitivity for miR-603/PEI complex compared to free miR-603	NA	NA	[399]
SNALPs	Anti-miR-21	CTX	NA	NA	Tumor size, apoptosis, angiogenesis and tumor cell proliferation	0.06 ± 0.07 fold change decrease in mir-21 levels compared to mismatch, 1.39 ± 0.24 fold change increase in RhoB and 53.7 ± 43.7 mm^3^ reduction in tumor size with anti-miR-21 + sunitinib in combination	[137]
SNA-liposomes and lipofectamine RNAimax	OMIs	ApoE and RVGpeptides	miR-92b downregulation in U87 GBM cell line	Downregulation of miR-92b expression using lipofectamine RNAimax (82 %), only SNA (65 %), SNA-liposomes (57 %), SNA-liposome-ApoE (78 %) with 23 % cell viability at 50 nm and 21 % at 25 nm,SNA-Liposome-RVG (86 %) and only liposome-ApoE with OMIs (50 %)	NA	NA	[483]
PAMAM and liposome	miR-7	FA	EGFR targeting miR-7 fragment transfection in U251 human glioma cells	31.79 ± 6.27 % reduction in migratory potential of miR-7 transfected cells	Survival study in mice	Increased mean survival duration from 16.4 ± 2.2 days to 23.5 ± 2.4 days post treatment with FA/PAMAM/miR-7	[484]
PLGA nanoparticles	Antisense miR-21	NA	miR-21 downregulation	15–20 % reduction in cell viability post treatment with 250 μM and 500 μM TMZ treatment in combination with 20 pmol of antisense miR-21	NA	NA	[256]
Branched PLA- PDMAEMA copolymer	miR-21 inhibitor	NA	miR-21 downregulation	79 % reduction in cell viability when ln229 GBM cells were treated with miR-21 inhibitor in combination with DOXO	Subcutaneous tumor growth inhibition in mice	Nine times reduction in tumor volume for miR-21 + DOXO (0.168 cm^3^) combination therapy compared to control group (1.52 cm^3^)	[257]
PAMAM based dendrimer	siLSINCT5	cell penetrating CendR peptide tLyp-1	Inhibition of LSINCT5-activated signaling pathways	Higher apoptosis (38.94 ± 1.68 %) observed in tLyp/aNKNP-siRNA treated U87 glioma cell compared to NP-siRNA (10.36 ± 0.81 %), NKNP-siRNA (12.13 ± 1.54 %), and tLypNP-siRNA (35.21 ± 0.88 %)	Survival study in mice	tLyp/aNKNP-siRNA treated group achieved the longest survival duration (>55 days) compared to other groups	[485]
MSNPs	Anti-miR-221	Cell penetrating R8 peptide	miR-221 inhibition	30 % cell viability in C6 glioma cells treated with TMZ and anti-miR-221 in combination and higher apoptosis (70.9 %) achieved in T98G cells treated with anti-miR-221 + TMZ in combination	NA	NA	[255]
SPIONs	si-HOTAIR	NA	Effect of deregulated lncRNA HOTAIR on human GSC tumorigenecity	Reduction of human GSC cell population in the S phase (12.06 ± 0.08 %) of cell cycle compared to an increase in cell population in G0/G1 phase (66.78 ± 0.96 %) and G2/M phase (21.17 ± 1.04 %)	Subcutaneous xenograft tumor growth inhibition in mice	Significant reduction in tumor size in tumors generated with human GSC cell population expressing low levels of HOTAIR	[451]
PMNP	anti-MALAT1 siRNA	NA	Targeting lncRNA MALAT1	50 % Knockdown of lncRNA MALAT1 using nanoparticle encapsulated si-MALAT1	Chemosensitization of intracranial GBM to TMZ in mice	Increased animal survival (>75 days) in animals treated with NP- anti-MALAT1 siRNA + TMZ in combination compared to control group	[452]

**SNALPs**: stable nucleic acid lipid particle; **CTX**: chlorotoxin; **SNA**: spherical nucleic acid; **OMIs**: oligonucleotide miRNA inhibitors; **ApoE**: apolipoprotein E; **RVG**: rabies virus glycoprotein; **PAMAM**: poly(amido amine); **FA**: folic acid; **EGFR**: epidermal growth factor receptor; **PLGA**: poly(lactic-co-glycolic acid); **PLA-PDMAEMA**: polylactic acid-polydimethylaminoethyl methacrylate; **MSNPs**: Mesoporous Silica Nanoparticles; **TMZ**: temozolomide; **DOXO**: doxorubicin; **SPIONs**: superparamagnetic iron oxide-based nanoparticles; **PMNP**: polymeric magnetite-bearing NP; **GSC**: glioma stem cells.

The absence of efficient carriers impairs the translation of miRNA therapy to clinical stages. In another study, Simion V. et al., formulated miRNA-Lipoplexes (LPRi) describing the kinetics of miRNA-133a after transfection of U-87MG GBM cells. They combined miRNA-ON reporter system (RILES) subcloned with Lentivirus expression vector (LentiRILES) for the analysis of miRNA uptake and functioning in transfected GBM cells. Herein, they implanted LentiRILES system for intracellular trafficking in the brain of mice and infused tumors with LPRi-miRNA. The live bioimaging revealed efficient delivery of miRNAs in GBM attesting internalization and activation of RISC signaling *in-vivo.* This experiment highlighted the potential of lipoplexes LPRI for miRNA-based therapy [425]. Grafals-Ruiz N. et al., functionalized gold nanoparticles with OMIs (oligonucleotide miRNA inhibitors) forming spherical nucleic acids (SNAs) complex. These SNAs were encapsulated into brain targeting ApoE (apolipoprotein E) peptide or RVG (rabies virus glycoprotein) conjugated liposomes forming SNA-liposome-ApoE or SNA-liposome-RVG lipoplexes. The internalization in U-87MG GBM cells *in-vitro* and intravenous administration in GBM syngeneic mice *in-vivo* showed downregulation of miR-92b, an aberrantly expressed miRNA in GBM cells or tumors. SNA-liposomes-ApoE showed better accumulation in tumor tissue as compared to control, or SNA-liposome, or SNA-liposome-RVG. This highlighted the promising role of SNA-liposome-ApoE for clinical translation of miRNA-based therapies for GBM or CNS disorders [426].

Recently, there are considerable number of reports proposing lipid nanocarriers as delivery systems coupling therapeutic miRNAs [427]. The small size, low molecular weight along with neutral charge represents efficient transfection strategy *in-vivo*. The core delivery system includes lipid-based nanocarriers but lacks specificity. In contrast, exosomes are an alternative option in regards to liposomes due to their biological similarity but they fail in clinical translation as they elicit a strong immune response. Therefore, the use of lipid-miRNA-based therapies as efficient nano delivery system is a challenging task concerning clinical platform.

#### 7.3.2 Polymeric nanocarriers

Polymeric nanocarriers have been classified depending upon their process of synthesis and preparation as nanospheres, nanoparticles and nano-capsules. Polymeric materials have emerged as promising vehicles for the delivery of small bio-macromolecules. Polymeric nanocarriers assembly is composed of structural variations consisting of hydrophobic core and hydrophilic shell formed by self-assembling properties of block-polymers [428]. They typically range from 100 to 200 nm in size, offer various advantages such as low cytotoxicity, high biocompatibility, sustained release, and also have ample surface area that can be functionalized with various targeting moieties [429,430]. Polymeric nanocarriers are constantly being developed and modified for improving the delivery of therapeutic cargo at the target site by both passive and active targeting methods. Polymeric nanocarriers offer unique advantages that can possibly overcome challenges owing to enhanced degree of porosity, high biocompatibility, superior pharmacokinetic and pharmacodynamic profile, and improved biodistribution *in vivo* [285,428]. Although polymeric nanocarriers holds several advantages they still have some major challenges such as prolonged circulation time, aggregation issues, protein corona formation, stability, toxicity and active targeting. These challenges can be tackled by modifying the method of synthesizing them, monomers used for their synthesis, surface modification etc. Further, polymeric nanocarriers are easily tunable which facilitates their ability to cross the BBB and effectively deliver the payload to target ncRNAs at tumor site [431].

##### 7.3.2.1 RNA-based therapeutics delivered using polymeric nanocarriers in GBM

In a study by Shatsberg Z et al., a redox-responsive polymeric nanogel was synthesized using inverse-nanoprecipitation method and used for the delivery of miR-34a, a tumor suppressor miRNA in a GBM mouse model [432]. Ectopic overexpression of miR-34a resulted in remarkable downregulation of its target genes that are involved in apoptosis and cell cycle arrest. When severe combined immunodeficient (SCID) mice bearing human U-87 MG tumors were treated with the nanogel-miR-34a (NG-miR-34a) and its respective control, it was seen that tumor progression was significantly halted in mice that were treated with NG-miR-34a as compared to the control. In another study, Poly (amido amine) PAMAM is reported to be an effective delivery vehicle for miR-7 into glioma cells because of low toxicity and prolonged release [433]. In a study, antisense-miR-21 was encapsulated inside PLGA nanoparticles and showed delayed release along with effective silencing function of miR-21 in GBM cells [434]. Also, co-delivery of doxorubicin encapsulated inside PLA (poly lactic acid) and poly dimethyl aminoethyl methacrylate conjugated with inhibitor of miR-21 resulted in inhibition of tumor progression as well as tumor volume reduction up to 9-fold suggesting efficient method of delivery by combination of chemotherapeutic drugs with miRNA therapy against GBM [435]. Similarly, the co-delivery of poly (amido amine) dendrimer conjugated miR-21 inhibitor along with 5-FU (fluorouracil) showed cytotoxicity in GBM cells [436]. Nanoparticles tagged with transferrin proved as efficient targeted delivery agent showing increased cytotoxicity against GBM cells when combined with zoledronic [437].

Apart from miRNAs, researchers have also targeted lncRNAs by delivering siRNAs against them using polymeric nanocarriers. Jin et al.*,* in 2020 developed a dual functioning dendrimer-based drug delivery system where they delivered siRNA against the long stress induced non-coding transcripts 5 (LSINCT5) lncRNA to GBM tumor. They functionalized PAMAM-based dendrimers using the cell penetrating peptide tLyp-1 that facilitated passage across the BBB, and the checkpoint inhibitor anti-NKG2A monoclonal antibody which helped in alleviating the immunosuppressive GBM microenvironment by activating both NK cells and T cells. This multimodal therapy effectively inhibited tumor progression in both *in vitro* and *in vivo* models of GBM [438].

#### 7.3.3 Lipid-polymer conjugates

From the name itself it is clear that lipid-polymer hybrid nanostructures are essentially core-shell nanoparticles derived from the combination of polymer and lipid where a core of polymer remains coated by lipid layer. Recently, their method of synthesis has been modified by using a synchronous one-way strategy where the polymer and lipid self-assemble, rather than a two-way strategy [439]. The dual property of lipid-polymer hybrid conjugates is especially useful for targeted cancer therapy, where the core can load a chemotherapeutic drug, while the surface lipid can be functionalized in variety of ways e.g., conjugating RNA, DNA, antibodies, and other targeting moieties [439].

Although the fusion mechanism is still unknown but the reason behind hybrid formation distinguishes the middle monolayer acting like a barrier limiting the transfer of entrapped drug restricting the degradation of core and water entry inside inner core. Majorly, structure is elucidated as drug containing polymer core-shell, lipid layer covering core and outer-lipid PEG layer for prolongation in the system [440]. The FDA approval was attained for lipid-polymer RNAi conjugate namely ONPATTRO™ by Alnylam Pharmaceuticals for treatment of Familial Amyloid Polyneuropathy. This approach as a therapeutic modality can be exploited further for targeted delivery of RNA-based anti-cancer agents in GBM.

##### 7.3.3.1 RNA-based therapeutics delivered using lipid-polymer conjugates in GBM

Shi et al. in 2015 synthesized modified RGD (Arginine-glycine-aspartic acid) lipid-polymer hybrid particles from Poly (Lactic-co-glycolic acid) PLGA-soy lecithin and 1, 2-Distearoyl-sn-glycero-3-phosphoethanolamine-Poly (ethylene glycol) DSPE-PEG for the delivery of docetaxel targeting GBM. The survival of rats bearing GBM tumor was improved drastically when treated with the above hybrid nanocarrier containing docetaxel as compared to control groups. It also facilitated enhanced cellular uptake, and penetration in tumor spheroids [441].

#### 7.3.4 Inorganic nanocarriers

These nanocarriers are developed using various inorganic materials such as metal, metal oxides and silica precursors. They offer several advantages in terms of drug delivery, e.g., their biocompatible nature, non-immunogenic, non-toxic, and easy to scale up properties. They can be suitably tailored owing to the advantage of controlled size, chemical stability and morphology. Silica-based nanocarriers such as silica nanoparticles or mesoporous silica nanoparticles have gained attention in clinical studies due to their high biocompatibility, biodegradability, and stability. Previous studies have reported the use of mesoporous silica nanoparticles to deliver antisense oligonucleotides for treating drug resistant cancers [255,442]. Similarly, nanoparticles developed from calcium phosphate are also inexpensive, non-toxic, and easy to synthesize having bioresorbable nature. During the last 40 years, they have been widely used for delivering nucleic acids into cells and helping in endosomal escape. They degrade easily in mild acidic environment of the lysozyme and release the payload in the cytoplasm [443]. In contrast, Iron oxide-based nanoparticles are extensively used for imaging purposes. They are used as contrast agents in MRI or drug and gene delivery. Magnetic nanocarriers consisting of cationic nanoparticles are favorable for higher encapsulation and efficacy of miRNA [444]. Another inorganic nanocarrier is gold nanoparticle, which have been used for almost a decade in drug delivery due to their virtue of tunable size, shape, functionalization and biocompatibility [[445], [446], [447]]. As discussed earlier in polymeric and lipid nanomaterials the challenges associated with a particular nanomaterial is based on several factors which involves different physicochemical properties of a material such as their shape, size, charge, surface moieties and most importantly material selection and the monomeric units involved. Similarly, the challenges with inorganic materials are also material specific which is based on material properties for example; the efficacy of gold nanoparticles is greatly dependent on their shape and size as it affects its surface plasmon resonance properties. Also, it is widely known that their shape and size can affect their biodistribution, toxicity, clearance by RES system and cellular interactions [448]. Similarly, different inorganic nanomaterials have different challenges associated with them depending on the material properties but one of the major concern is their in-vivo fate which is important for their clinical translation.

##### 7.3.4.1 RNA-based therapeutics delivered using inorganic nanocarriers in GBM

Bertucci et al. in 2015 synthesized mesoporous silica nanoparticles (MSNPs) targeting drug resistant GBM by co-delivering PNA anti-miR-221 conjugated with octaaarginine and TMZ. Studies report upregulation of miR-221 promotes progression of tumor growth and chemoresistance in glioma cells. Therefore, co-delivery of anti-miR-221 and TMZ showed an induction of apoptosis in the TMZ resistant T98G human glioma [255]. Magnetic nanoparticles have shown advantage in gene therapy for miRNA delivery. In 2014, Yin et al. synthesized a highly magnetic zinc doped iron oxide nanoparticles ZnFe_2_O_4_ for the delivery of tumor suppressor miRNA let-7a through magnetic hyperthermia. While let-7a targeted the genes involved in the heat shock response, an alternating magnetic field was applied to generate heat that ultimately led to apoptosis of GBM cells [449]. Also, in 2014 Yang et al. developed a PAMAM (poly-amidoamine) grafted, Gd (gadolinium) functionalized nanographene oxide (NGO) carrier that effectively delivered the miRNA let-7g, along with epirubicin (EPI), a chemotherapeutic drug to U-87 MG GBM cells. This nanocomplex not only inhibited tumor progression by targeting the RAS family of oncogenes, but could also be exploited as a contrast agent in magnetic resonance imaging (MRI) technique [450].

Fang et al.*,* in 2016 developed a superparamagnetic iron oxide-based nanoparticle to deliver siRNA against the lncRNA HOTAIR to GBM tumor using magnetofection technique. This resulted in a marked reduction of cellular proliferation and invasion of GBM cells (Table 3) [451]. In 2019, Voce et al.*,* also demonstrated the use an iron oxide-based core-shell nanocarrier to deliver siRNA against the lncRNA MALAT1 along with TMZ to mice bearing intracranial xenograft of GBM. This sensitized the GBM cells towards TMZ treatment (Table 3) [452]. Sukumar U.K. et al., in 2019, developed inorganic nanocarriers of gold and iron oxide nanoparticles referred to as GIONS, co-functionalized with hybrid polymer (β-cyclodextrin-chitosan) and PEG-T7 peptide for intranasal delivery. These GIONS were developed for the co-delivery of miR-100 and anti-miR-21 along with TMZ. Their strategy of developing polyfunctional nanoparticles loaded with therapeutic miRNAs exhibited synergistic effects in orthotopic GBM xenografts in mice [453].

#### 7.3.5 Metal organic frameworks (MOFs)

With the recent development of complex materials in nanotechnology over the last two decades, cancer therapeutics have gained attention as par efficiency in early diagnosis and therapy. Metal organic frameworks (MOFs) are hybrid materials having both organic and inorganic (metal) components. Typically, these are cage like structures where an array of positively charged metal ions are linked through organic molecules. By varying the nature of metal ions and linker molecules, MOFs can be utilized for a variety of applications, ranging from gas storage to drug delivery [454,455]. Recently, a series of porous crystalline inorganic-organic hybrid materials composed of metal ions, clusters or metallic ligands are scaled up to nano-size referred to as nanoscale MOFs [456]. These are currently used as efficient delivery systems due to many advantages such as high loading efficiency, sustained and controlled drug release, high accumulation rate at tumor site, biocompatibility and biodegradability. Apart from these they sustain as attractive theranostic platforms in delivering imaging contrast agents or in chemotherapeutics. The major drawback of MOFs is the premature release of therapeutic drugs but in relation to delivery of nucleic acids there are only limited studies, thus it's difficult to conclusively comment on their limitations till they are validated in the preclinical and clinical models.

##### 7.3.5.1 RNA-based therapeutics delivered using MOFs as nanocarriers for GBM

Till date no major studies exist that involves the delivery of therapeutic oligonucleotides to GBM using MOFs but several reports exist which involves the development of MOFs for chemotherapeutics delivery to GBM. However, very recently in 2022, Liang, D et al., reported the development of organic frameworks referred to as “magnetic covalent organic framework (MCOF)” was developed with iron oxide core and organic framework as shell as a biosensor for the detection of miR-182 from blood samples of glioma patients [457].

#### 7.3.6 Nucleic acid as nanocarriers

In comparison to several other organic and inorganic nanocarriers developed in the past, nucleic acids as carriers hold several advantages such as excellent biocompatibility, programmability, low toxicity, size homogeneity and controlled response to intrinsic biological environment [458,459]. Nucleic acid nanocarriers as a distinct class of nanostructures consist of DNA, RNA, both or just simple assemblies made from synthetic oligo's [459]. The nucleic acid nanostructure assembly is simple based on the Watson and Crick model of complementary base pairing. Therefore, it easily allows the construction of controllable nanostructures with just a simple tweaking in base pairs [459,460]. Several nucleic acid nanocarriers have been synthesized till date which are mainly categorized as either self-assembled nucleic acid nanocages or organic and inorganic nanoparticles decorated with nucleic acids. Within this broader category, it includes DNA nanocages, DNA prism, DNA tetrahedron, gemcitabine (analog of deoxycytidine)-based self-assembled nanocomplexes, DNA/RNA composites and DNA/RNA hybrids etc [[460], [461], [462], [463], [464], [465], [466], [467], [468]]. These nucleic acid nanostructures can not only avoid renal clearance but can also associate them with the EPR effect within the tumor vasculature apart from having better nuclease stability and cellular uptake [[469], [470], [471], [472]]. Thus, nucleic acids as nanocarriers offer a smart strategy when it comes to co-deliver drugs such as small molecules and nucleic acids as cargo to treat GBM. Nanocarriers designed using macromolecules such as nucleic acids are in very nascent stages of their development. Because of the complexity in their structure, it could be difficult to predict their outcome in in-vivo system which could hamper the clinical translation. Thus, it is essential to fully understand their pharmacokinetics before translating them to clinics.

##### 7.3.6.1 RNA-based therapeutics delivered using nucleic acid nanocarriers in GBM

In 2021, Kumthekar et al. developed spherical nucleic acids consisting of siRNA conjugated gold nanoparticle core targeting *Bcl2L12* (NU-0129) in GBM. After successfully conducting pre-clinical studies in non-human primates, the group conducted the first successful phase 0 clinical trials in human (NCT03020017) [403,473]. Kumthekar et al. were able to establish that by delivering siRNAs using SNA nanocomposites targeting the oncogene Bcl2Like12 in GBM was not only a precision medicine approach to treat GBM but acted as a smart strategy by crossing the BBB for targeted therapy [403]. Gao et al., in 2021, developed nucleic acid nanogel that mimics the viral membrane structure for the delivery of miR-155 to reprogram the microglia and macrophages from an invasive anti-inflammatory M2 phenotype to pro-inflammatory M1 phenotype. The DNA nanostructure was designed using complementary overhangs corresponding to miR-155 to act as a crossslinker for nucleic acid grafted polymer brush that was further coated with M2 and HA2 peptides embedded in erythrocyte membrane to develop a virus mimicking structure. With this approach, the group was successfully able to develop the immunotherapy-based approach to treat GBM by modulating the functions of immune cells such as microglia and macrophages [474].

#### 7.3.7 Proteins and peptide-based nanocarriers

Proteins and peptides are nature's tool that has an intrinsic ability to self-assemble into defined nanostructures. Both being composed of amino acids as monomeric units with amide bonds when assembled as a nanocarrier display excellent biological properties such as cell membrane penetration and endosomal escape [[475], [476], [477], [478]]. They are not only biocompatible and biodegradable but because of their amphiphilic nature, they can easily interact with different cargos used as therapeutics to treat numerous diseases [479]. Proteins or peptide nanocarriers have an added advantage over several other synthetically designed nanocarriers that includes structural stability, uniform size and shape distribution and easy access to internal and external subunits for funtionalization. Depending upon the source and composition of protein and peptides, they can be broadly classified as 1) Natural structure-based nanocarriers such as ferritins, small heat shock proteins (sHsp) and vaults; 2) Virus like particle-based nanocarriers which includes self-assembled viral protein coats extracted from different viruses such as cowpea chlorotic mottle virus, cowpea mosaic virus, bacteriophage MS2, hepatitis B virus, red clover necrotic mosaic virus, avian sarcoma leukosis virus, hibiscus ringspot virus and rotavirus VP6 etc.; and 3) de novo designed protein and peptide nanocarriers using computational tools to symmetrically arrange the protein blocks into a self-assembling nanoarchitecture [476,480]. Protein nanocarriers are considered as non-toxic and safer alternative compared to several other categories of nanocarriers owing to its intrinsic biological properties however, in some cases, protein nanocarriers are reported to induce the expression of inflammatory markers [479,481]. But overall, protein-based nanocarriers are promising delivery agents of delivery of RNA, DNA, and other chemotherapeutic drugs to GBM tumors.

##### 7.3.7.1 RNA-based therapeutics delivered using protein nanocarriers in GBM

In a recent study, Mondal et al. has developed PEI functionalized transglutaminase-based nanoflowers (TGNFs) to deliver anti-miRs to GBM cells. They have shown that delivering anti-miR-210 to GBM cells using these TGNFs significantly inhibits tumor progression by decreasing cellular proliferation, migration, and inducing apoptosis [482]. In 2020, Gregory et al. demonstrated efficient delivery of siRNA molecules using human serum albumin (HSA)-based synthetic protein nanoparticles (SPNP) to intracranial GBM [404]. The SPNP comprised of polymers of HSA, and oligo (ethylene glycol) (OEG); while the cyclic peptide iRGD (containing Arg-Gly-Asp motif) was used for targeted delivery. They successfully delivered siRNA against the oncogene signal transducer and activator of transcription 3 (si-STAT3) to the highly aggressive intracranial GBM tumor via non-invasive systemic administration. The SPNP efficiently crossed the highly selective BBB of GBM and delivered the cargo. Combining this RNAi approach with focused radiotherapy, they could achieve high survival rate of 87.5 % of tumor bearing mice. Their strategy suggested synthetic protein-based nanocarrier as an effective delivery vehicle along with STAT3i an advantageous moiety providing immunomodulatory response in aggressive recurring GBM. This study also highlighted the potential of the tissue penetrating, and tumor targeting cyclic peptide iRGD as a targeting agent for GBM that binds to the αvβ3 and αvβ5 integrins overexpressed in tumor vasculature.

#### 7.3.8 Vector mediated nucleotide therapy in clinical trials

Numerous strategies are being implied to tackle down a never-ending quest for treating GBM. Few nucleotide-based therapeutics that are delivered to tumor site using viral & non-viral vectors are undergoing clinical trial. The clinical data has been tabulated in Table 4 as per data accessed through https://clinicaltrials.gov/.

**Table 4:** Recent clinical trial data for viral & non-viral vector mediated nucleic acid therapy to treat GBM.

S.No.	Vector/Nucleotide	Mechanism of action	Participants	Phase of trial and ID	Recruitment status	Completion
1)	RNAi-based SNA (NU-0129) as targeted molecular therapy	Delivery of nucleic acid targeting *Bcl2L12* gene associated with apoptosis in GBM.	8	Early phase I (NCT03020017)	Completed	Completed
2)	RNA-LP vaccines	Delivery of RNA loaded lipid nanoparticles against newly diagnosed unmethylated *MGMT* in adult GBM.	28	Phase I (NCT04573140)	Recruiting	July 2027
3)	CMV RNA pulsed DC vaccines	Human CMV matrix protein pp65-flLAMP coding RNA loaded in autologous dendritic cells against newly diagnosed GBM patients undergoing SOC treatment.	10	Phase I (NCT04963413)	Recruiting	May 2025
4)	CMV RNA pulsed DC with Td toxoid vaccine	Immunotherapy approach targeting pp65 antigen against newly diagnosed GBM.	175	Phase II (NCT02465268)	Recruiting	June 2024
5)	Lentiviral vector expressing human interferon-alpha 2 gene	Lentiviral vector mediated expression of interferon-alpha2 against GBM patients with unmethylated MGMT promoter.	27	Phase I/II (NCT03866109)	Recruiting	December 2024
6)	SGT-53 + TMZ	Cationic liposomal delivery of *wild type p53* gene sequence in a plasmid as a backbone to restore the function of p53 tumor suppressor gene in combination with TMZ for patients with recurrent GBM.	1	Phase II (NCT02340156)	Terminated	November 2018
7)	CART-EGFRvIII (lentiviral vector expressing EGFRvIII)	Lentiviral vector mediated expression of chimeric antigenic receptor against patients with recurrent EGFRVIII + GBM.	11	Phase I (NCT02209376)	Terminated	April 2018
8)	Adenoviral vector expressing hIL-12	Adenovirus vector mediated expression of human IL-12 in combination with veledimex as an immunotherapy approach for patients with recurrent GBM.	40	Phase I (NCT02026271)	Completed	August 2019
9)	ADV/HSV-tk	Viral vector mediated delivery of HSV-tk gene along with nucleoside analog, valacyclovir in combination with radiation therapy leading to immunogenic cell death for recurrent GBM.	62	Phase I/II (NCT03596086)	Recruiting	December 2025

**SNA**: spherical nucleic acid; **RNA-LP**: RNA-lipid particles; **CMV**: cytomegalovirus; **DC**: dendritic cells; **Td**: Tetanus and Diphtheria; **CART-EGFRvIII**: chimeric antigen receptor T cells-epidermal growth factor receptor variant III; **hIL-12**: human interleukin-12; **ADV/HSV-tk**: adenovirus/herpes simplex virus-thymidine kinase.

## 8 Authors perspective

Although there have been significant advancements in the fields of biomedicine and cancer biology, patients diagnosed with GBM still have a poor prognosis, with an average survival rate of only 15 months post-diagnosis that has not improved in the last 30 years. Despite various clinical trials being conducted, only a handful of them have received FDA approval. Consequently, the prognosis for GBM patients is still primarily dependent on conventional treatments. This indicates a significant gap in translating research findings from the laboratory to the clinical setting.

RNA-based molecular therapy holds tremendous advantages over conventional drug therapy as it is target-specific. They mainly target the transcripts of mRNAs, miRNAs, lncRNAs, circRNAs, piRNAs, NATs and several other RNAs leading to their degradation or inhibition of translation. The selection and identification of suitable RNA targets, dose requirement and efficacy, administration schedule and, more likely, a validated response can promise future clinical strategies for targeting GBM. The success of RNA-based molecular therapy for GBM requires an interdisciplinary collaborative approach with advancements in nanomedicine, molecular medicine, pharmacology, neurobiology, and immunology for a targeted therapeutic outcome. There has always been a misconception and ambiguity about the roles played by ncRNAs in how they regulate different physiological, biological, and pathological processes. Compared to a common notion where they have been considered junk, ncRNAs are now known to play a crucial role in regulating different hallmarks of cancer development such as cellular proliferation, migration, apoptosis, angiogenesis, stemness., etc. Not just independently, but a matrix of different ncRNAs-mRNA signaling axis plays a role in GBM tumorigenesis. Identifying and sequentially targeting these ncRNA using synthetic oligonucleotides can disrupt the whole regulatory network that regulates different cancer hallmarks to promote tumorigenesis. Moreover, combining RNA-targeting therapies with conventional treatments such as surgery, chemotherapy and radiotherapy may enhance their effectiveness. Thus, RNA-based molecular therapy holds the potential to significantly improve GBM patients’ prognosis, but it has its own challenges and must be carefully met before considering their translation to clinics.

The major challenge is the BBB-BTB-TME triad which orchestrates the pathophysiology of GBM. The failure to surpass this triad has been a significant cause of numerous clinical trial studies' failure, indicating its clinical significance in designing RNA therapy for GBM. In addition, serum nucleases present another significant hurdle, as they can degrade the therapeutic RNA molecules even before they reach the GBM tissue penetrating the BBB-BTB-TME triad. Carefully engineered smart nanomaterials with different physicochemical properties and surface-modified nanomaterials for active targeting of GBM can not only protect the therapeutic RNA cargo from nucleases but can also help them to cross the BBB-BTB-TME triad for their successful delivery to the brain. The nanomaterials should be designed considering their pharmacokinetics and pharmacodynamics in the biological system, such as the biodistribution patterns, systemic toxicity, cellular uptake, and endosomal escape, which may contribute to undesired outcomes if not carefully considered leading to therapy failure. Various kinds of inorganic, organic, and biological nanocarriers have been developed over the time to circumvent these problems. Each of these has its own advantages and disadvantages. However, in our opinion, nanomaterials with inherent biological properties that can easily evade first pass metabolism, modulate EPR effect, have prolonged circulation time, protect the RNA-cargo from serum nucleases, discourage replacement by other polyanion moieties, and evade immune response from the RES system are deemed to be the ideal carriers for RNA therapeutic payload delivery.

Despite the tremendous advancement, RNA-based nanotherapeutics still face strong challenges to completely eradicate GBM. The problem not only lies in the dearth of effective delivery agents, but the choice of the therapeutic RNA is also crucial. As previously mentioned, targeting a single miRNA or lncRNA might not be enough as the cancer cells can soon learn ways to bypass it. Also, tumor heterogeneity is a major issue. Every GBM patient has a unique genetic signature and the mutations one accumulates might be different from another. So, GBM therapy should become more personalized in nature, where the deregulated RNA or ncRNA signature of a patient should be identified first via high throughput sequencing techniques, and a multimodal therapy should be designed accordingly.

## 9 Conclusion

Considering the diverse roles played by ncRNAs in regulating different hallmarks in GBM tumorigenesis, it is essential to not only focus on understanding and targeting a single ncRNA but an array of network and sequencing analysis must be done to understand their complex biology. Several studies have reported that targeting ncRNA using synthetic oligonucleotides has greatly reduced the tumor volume in animal studies but translating it to clinics is still a daunting task which is evident from the limited studies in clinical trials targeting ncRNAs in GBM. This is mainly due to their failure involving multitude of factors after their administration in the biological system such as degradation by serum RNAses, rapid clearance by reticuloendothelial system, improper dosage, non-specific targeting and most importantly the BBB-BTB-TME triad. Such challenging issues can be addressed by engineering smart nanomaterials that can intermediate to increase the therapeutic response of ncRNA therapy in their translation from bench to bedside.

## CRediT authorship contribution statement

**Ravi Raj Singh:** Conceptualization, Data curation, Formal analysis, Investigation, Methodology, Writing – original draft, Writing – review & editing. **Indranil Mondal:** Investigation, Writing – review & editing. **Taskeen Janjua:** Writing – review & editing. **Amirali Popat:** Conceptualization, Project administration, Resources, Supervision, Writing – review & editing. **Ritu Kulshreshtha:** Conceptualization, Project administration, Resources, Supervision, Writing – review & editing.

## Declaration of competing interest

The authors declare that they have no known competing financial interests or personal relationships that could have appeared to influence the work reported in this paper.
