# Supplementary material for: Engineered smart materials for RNA based molecular therapy to treat Glioblastoma
Source: Bioact Mater. 2023 Nov 27;33:396–423. doi: 10.1016/j.bioactmat.2023.11.007 (PMC10696434; doi:10.1016/j.bioactmat.2023.11.007)
Supplement: Multimedia component 1 [file mmc1.docx]

List of genes/proteins mentioned in abbreviation in the main manuscript

1. Notch receptor 1 and notch receptor 2 (NOTCH1 and NOTCH2)
2. Platelet-Derived Growth Factor Receptor Alpha (PDGFRA)
3. Silent information regulator 1 (SIRT1)
4. MET proto-oncogene (MET)
5. RNA-binding protein Musashi1 (MSI1)
6. B lymphoma mouse Moloney leukemia virus insertion region 1 (Bmi-1)
7. Suppressor of zeste 12 homolog–Drosophila (SUZ12)
8. Urokinase-type plasminogen activator receptor (uPAR)
9. Ras Homolog Family Member C (RhoC)
10. Homeobox D10 (HOXD10)
11. Cyclin dependent kinase inhibitor 2A (CDKN2A)
12. Cyclin dependent kinase inhibitor 1A (CDKN1A)
13. Transcription Factor AP-2γ (TFAP2C)
14. Leucine rich repeat (in FLII) interacting protein 1 (LRRFIP1)
15. Heterogeneous nuclear ribonucleoprotein K (HNRPK)
16. Programmed cell death 4 (PDCD4)
17. Phosphatase and tensin homologue (PTEN)
18. Transforming growth factor-β (TGF-β)
19. Acidic nuclear phosphoprotein 32 family, member A (ANP32A) (alias pp32 or LANP)
20. SWI/SNF-related, matrix-associated, actin-dependent regulator of chromatin, subfamily a, member 4 (SMARCA4)
21. Matrix metalloproteinases (MMPs)
22. Heterogeneous nuclear ribonucleoprotein K (HNRNPK)
23. Homeobox C (HOXC)
24. hypoxia-inducible factors 1α (Hif-1α)
25. NKD Inhibitor of WNT Signalling Pathway 1 (NKD1)
26. Leucine-rich repeats and immunoglobulin-like domains 1 (LRIG1)
27. E2F Transcription Factor 6 (E2F6)
